# Supplementary material for: Protein–protein and protein–nucleic acid binding site prediction via interpretable hierarchical geometric deep learning
Source: Gigascience. 2024 Nov 1;13:giae080. doi: 10.1093/gigascience/giae080 (PMC11528319; doi:10.1093/gigascience/giae080)
Supplement: giae080_GIGA-D-24-00255_Original_Submission [file giae080_giga-d-24-00255_original_submission.pdf]

GigaScience

Protein-protein and protein-nucleic acid binding site prediction via interpretable hierarchical geometric deep learning

--Manuscript Draft--

**Manuscript Number:**	GIGA-D-24-00255
**Full Title:**	Protein-protein and protein-nucleic acid binding site prediction via interpretable hierarchical geometric deep learning
**Article Type:**	Technical Note
**Funding Information:**	National Key R&D Program of China (2020YFA0712400) — Dr. Juntao Liu<br>National Natural Science Foundation of China (62272268) — Dr. Juntao Liu
**Abstract:**	Identification of protein-protein and protein-nucleic acid binding sites provides insights into biological processes related to protein functions and technical guidance for disease diagnosis and drug design. However, accurate predictions by computational approaches remain highly challenging due to the limited knowledge of residue binding patterns. The binding pattern of a residue should be characterized by the spatial distribution of its neighboring residues combined with their physicochemical information interaction, which yet can not be achieved by previous methods. Here, we design GraphRBF, a hierarchical geometric deep learning model to learn residue binding patterns from big data. To achieve it, GraphRBF describes physicochemical information interactions by designing an enhanced graph neural network and characterizes residue spatial distributions by introducing a prioritized radial basis function neural network. After training and testing, GraphRBF shows great improvements over existing state-of-the-art methods and strong interpretability of its learned representations. Applying GraphRBF to the SARS-CoV-2 omicron spike protein, it successfully identifies known epitopes of the protein. Moreover, it predicts multiple potential binding regions for new nanobodies or even new drugs with strong evidence. A user friendly online server for GraphRBF is freely available at http://liulab.top/GraphRBF/server.
**Corresponding Author:**	Juntao Liu<br>Shandong University<br>Weihai, CHINA
**Corresponding Author Secondary Information:**	
**Corresponding Author's Institution:**	Shandong University
**Corresponding Author's Secondary Institution:**	
**First Author:**	Shizhuo Zhang
**First Author Secondary Information:**	
**Order of Authors:**	Shizhuo Zhang<br>Jiyun Han<br>Juntao Liu
**Order of Authors Secondary Information:**	
**Additional Information:**	
**Question**	**Response**
Are you submitting this manuscript to a special series or article collection?	No
**Experimental design and statistics**	Yes

Powered by Editorial Manager® and ProduXion Manager® from Aries Systems Corporation

Full details of the experimental design and statistical methods used should be given in the Methods section, as detailed in our Minimum Standards Reporting Checklist. Information essential to interpreting the data presented should be made available in the figure legends.<br><br>Have you included all the information requested in your manuscript?	
**Resources**<br><br>A description of all resources used, including antibodies, cell lines, animals and software tools, with enough information to allow them to be uniquely identified, should be included in the Methods section. Authors are strongly encouraged to cite Research Resource Identifiers (RRIDs) for antibodies, model organisms and tools, where possible.<br><br>Have you included the information requested as detailed in our Minimum Standards Reporting Checklist?	Yes
**Availability of data and materials**<br><br>All datasets and code on which the conclusions of the paper rely must be either included in your submission or deposited in publicly available repositories (where available and ethically appropriate), referencing such data using a unique identifier in the references and in the "Availability of Data and Materials" section of your manuscript.<br><br>Have you have met the above requirement as detailed in our Minimum Standards Reporting Checklist?	Yes

Manuscript

Click here to access/download;Manuscript;Manuscript of GraphRBF.pdf

Protein-protein and protein-nucleic acid binding site prediction via interpretable hierarchical geometric deep learning

Shizhuo Zhang[1,†], Jiyun Han[1,†], Juntao Liu[1,*]

[1]School of Mathematics and Statistics, Shandong University (Weihai), Weihai, 264209, China

[†]These authors contributed equally to this study

[*]To whom correspondence should be addressed

Email addresses:

JL: juntaosdu@126.com

Abstract

Identification of protein-protein and protein-nucleic acid binding sites provides insights into biological processes related to protein functions and technical guidance for disease diagnosis and drug design. However, accurate predictions by computational approaches remain highly challenging due to the limited knowledge of residue binding patterns. The binding pattern of a residue should be characterized by the spatial distribution of its neighboring residues combined with their physicochemical information interaction, which yet can not be achieved by previous methods. Here, we design GraphRBF, a hierarchical geometric deep learning model to learn residue binding patterns from big data. To achieve it, GraphRBF describes physicochemical information interactions by designing an enhanced graph neural network and characterizes residue spatial distributions by introducing a prioritized radial basis function neural network. After training and testing, GraphRBF shows great improvements over existing state-of-the-art methods and strong interpretability of its learned representations. Applying GraphRBF to the SARS-CoV-2 omicron spike protein, it successfully identifies known epitopes of the protein. Moreover, it predicts multiple potential binding regions for new nanobodies or even new drugs with strong evidence. A user friendly online server for GraphRBF is freely available at http://liulab.top/GraphRBF/server.

Introduction

Interactions between proteins and nucleic acids are fundamental to a myriad of biological activities and processes, including gene replication, expression, signal transduction, regulation, and metabolism[1-3]. These interactions are the cornerstone of most protein functions and are essential for analyzing genetic material, elucidating protein functions, and facilitating drug design[4,5]. However, experimental methods such as X-ray crystallography, NMR spectroscopy, and affinity purification are often costly and time-intensive, highlighting the urgent need for reliable and accurate computational methods capable of large-scale prediction of protein functional sites[6,7]. Existing computational methods can be generally categorized into two groups: sequence-based methods and structure-based methods. Sequence-based methods, such as SCRIBER[8], TargetDNA[9], DRNApred[6] and hybridRNAbind[10], use only protein sequences to predict protein interaction sites. The advantage of sequence-based methods is the rapid prediction of binding sites using only the sequence information of the protein. However, these methods ignore the most important information of protein spatial structure, which directly determines its function[11]. Structure-based methods use both the sequence and structural information of proteins and analyze proteins based on their 3D structures, such as GraphBind[12], ScanNet[13] and MaSIF-site[14].

Deep learning models can learn data features and their invariants directly through back propagation and have stronger generalization capabilities. Many deep learning models have been used for predicting protein binding sites in recent years, such as Convolutional Neural Network(CNN)[15], Transformer[16], Graph Neural Network(GNN)[12,17], and geometric deep learning[13,14]. For example, Spatom[17] combines graph convolutional neural networks and graph attention networks to predict protein interaction sites. GraphBind[12] extracts graph features based on the local structural context of amino acids using a graph neural network for nucleic acid binding site prediction. ScanNet[13] learns features directly from the 3D structure by constructing spatio-chemical arrangement of neighboring atoms and amino acids for an atom and amino acid, and then predicts hierarchical representations using amino acid features. MaSIF-site[14] uses geometric deep learning to capture protein surface fingerprints, which are then classified using a convolutional network.

The function of a protein is mainly determined by the structure of the protein, especially the localized pattern of the tertiary structure[18]. Adapting deep learning methods to protein structures requires appropriate descriptions of proteins followed by comprehensive feature extractions and message passing according to protein 3D structures. There are various structural descriptions of proteins, such as residue graphs[12,17,19], atomic point clouds[20], and molecular surfaces[14]. The graph description approach involves using protein residues as nodes and the distances between them as edges to derive a graph from the 3D structure. Graph descriptions are not only invariant to rotation and translation, but can also handle different

numbers of disordered neighbors of residues and preserve the topological relationships between residues[12]. However, graph-based methods can only rely on the graph structure for node and edge feature updating, rather than feature extraction based directly on real 3D spatial distributions, such as coordinates. Point cloud-based methods can directly use the 3D coordinates of each point to extract features and then analyze their spatial distribution, which is helpful for us to understand the real spatial distribution of protein residues[20-22]. However, point cloud-based methods overlook the interactions and information exchange between amino acid nodes, leading to a loss of association information between residues. Surface-based methods can intuitively represent the surface properties of proteins, facilitating the understanding of interactions between proteins and their ligands[14,23]. However, they depend on high-precision protein surface models, which often require significant computational resources. At the same time, surface-based methods also overlook the impact of internal residues and the complete local structure of the protein on the determination of binding sites.

In this study, we introduce GraphRBF, an interpretable hierarchical geometric deep learning framework for accurately identifying binding sites by comprehensively characterizing residue binding patterns encompassing physicochemical information interactions and residue spatial distributions. GraphRBF first extracts local neighbors for each residue and constructs a local graph with nodes representing residues and edges denoting their close distances. Then, an enhanced graph neural network is developed to learn physicochemical information interactions in the local graph and a prioritized radial basis function neural network is designed to capture the local residue spatial distributions. In this study, a new radial basis function (termed gentle decay Gaussian-like function) is introduced to better adapt to the framework and a kernel attention mechanism is built to prioritize the learned representations from different kernels. Finally, residues can be classified into binding or non-binding sites by combining all the learned local graph representations.

After a comprehensive evaluation, GraphRBF demonstrates great improvements over existing state-of-the-art methods on three protein binding site prediction tasks, including predictions for protein-protein, protein-DNA and protein-RNA binding sites. In addition, we found that GraphRBF shows strong interpretability by visualizing and analyzing its learned kernel functions and high-level representations, as well as their relationships with important physicochemical features of protein binding sites. We also applied GraphRBF on a new protein structure, the Severe Acute Respiratory Syndrome Coronavirus 2 (SARS-CoV-2) spike protein, and found that the model's predictions for the NTD, RBD, SD1, and SD2 regions reveal high scores, completely consistent with known antibody binding sites. Notably, the NTD's flexibility and the RBD's role in ACE2 receptor binding make these two regions significant targets for neutralizing antibodies. Moreover, GraphRBF identifies multiple

potential binding regions for new nanobodies or even new drugs with strong evidence from a comprehensive analysis. These findings may contribute to the understanding of the spike protein's antigenic properties and interaction with the immune system, which are vital for vaccine and therapeutic development.

Results

The GraphRBF framework

The overall framework of GraphRBF, shown in Fig. 1, can be broadly divided into four parts: (1) extraction of raw residue features and their coordinates; (2) construction of a local environment for each residue by collecting its spatial neighbors; (3) comprehensive representation of each local environment by combining an enhanced graph neural network and a prioritized radial basis function neural network; (4) generation of predicted binding probabilities (Fig. 1a).

The node features are extracted based on the protein sequence and structure for each amino acid, while the residue coordinates are defined by the 3D coordinates of its α-carbon. The sequence based features comprise evolutionary information from position-specific score matrix (PSSM), hidden Markov model profile (HMM), and one-hot of residue types (RT), and the structure based features include secondary structure information (DSSP) and atom features (AF). The coordinates of the residue are directly extracted from the input structural file.

Towards learning the binding patterns of a residue from its local neighborhood, we first extract its spatial neighbors and construct a local coordinate system that ensures rotation and translation invariance and then we build a local graph with nodes representing residues and edges denoting their close distances (Fig. 1b).

To capture the physicochemical relationships among the target residue and its neighbors, we construct an Enhanced Graph Neural Network (EGNN) to capture the positional and physicochemical information of neighboring residues by the message passing mechanism and update the features of nodes and edges accordingly in the local graph. To extract the spatial distributions of its neighbors, we also design a Prioritized Radial Basis Neural Network (PRBFNN) and propose a Gentle Decay Gaussian-like kernel function (GDG function) used in our spatial filters to delineate the spatial distribution of the local neighborhood and learn its representation as the local graph feature (Fig. 1c). A kernel attention mechanism is applied to prioritize the learned representations from different kernels. This process is iterated K times, resulting in the generation and concatenation of K graph features into a classification vector.

Finally, in order to highlight the important elements of all graph features and classify whether the central residue is a binding site or not, we apply the attention mechanism to

dynamically weight each dimension of the classification vector, followed by a residue classification through a multilayer perceptron.

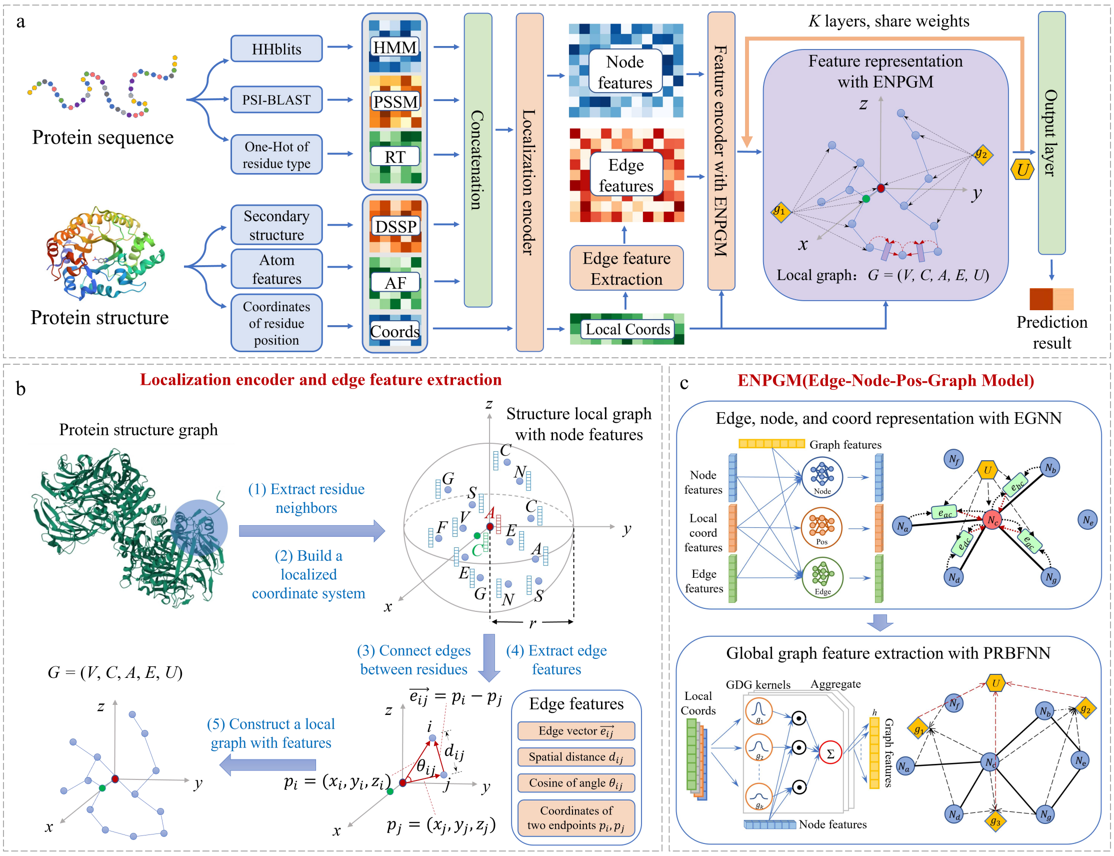

Fig. 1 The framework of GraphRBF. **a,** Overview of GraphRBF. The inputs of the model mainly contain PSSM, HMM and One-Hot encoding of amino acid types based on sequence, DSSP and atomic features based on structure, and coordinates of residue position. After the concatenation of features and localization of nodes with their coordinates, we extract the edge features and build the local graph. These features are further encoded and represented by the ENPGM to obtain the graph features. And the output layer use these graph features to get the final prediction results. **b,** the localization encoder and edge feature extraction module of GraphRBF extracts the neighborhood and constructs the localized coordinate system. Then the edge vectors, spatial distances and cosine of angles and coordinates of two endpoints are used as edge features, and finally the graph representation G = (V, C, A, E, U) is obtained, where V is the node feature set, C is the localized coordinate set, A is the adjacency matrix, E is the edge feature set and U is the graph feature set. **c,** The two sub-modules of the Edge-Node-Pos-Graph Model (ENPGM) includes an Enhanced Graph Neural Network (EGNN) and a Prioritized Radial Basis Function Neural Network (PRBFNN) based on GDG function kernels.

Comparison with state-of-the-art methods on benchmark datasets

In this section, we compare the performance of our proposed model with several state-of-the-art models using the evaluation metrics such as Recall (Rec), Precision (Pre), F1-Score (F1), Matthews Correlation Coefficient (MCC), Area Under the Receiver Operating Characteristic Curve (AUROC), and Area Under the Precision-Recall Curve (AUPRC) (see Supplemental Method S1). In the protein-protein binding site test set, we compared GraphRBF with the structure-based models SPPIDER[24], GraphPPIS[25], MaSIF-site[14] and ScanNet[13]. In the protein-nucleic acid binding site prediction task, we compared GraphRBF with both the sequence-based models TargetDNA[9], hybridRNAbind[10], DeepDISOBind[26] and DRNApred[6], and the structure-based models GraphBind[12]. The comparison is performed on the levels of the entire test sets and individual proteins in each test set.

Comparison on the three entire test sets. The results of the performance comparison with the above methods on the corresponding test sets are displayed in Table 1. On the protein-protein binding test set, the AUC, PRC, F1-score, and MCC of GraphRBF are 0.028, 0.108, 0.055, and 0.066 higher than the second highest value, with relative improvements achieving 3.5%, 41.9%, 16.2%, and 24% respectively. On the protein-DNA (RNA) binding test set, the AUC, PRC, F1-score, and MCC of GraphRBF are 0.017 (0.076), 0.052 (0.106), 0.061 (0.087), and 0.049 (0.095) higher than the second highest value, with relative improvements reaching 1.9% (9.2%), 18.0% (26.1%), 17.4% (20.0%), and 13.8% (24.7%), respectively.

The superiority of GraphRBF over SPPIDER and GraphPPIS demonstrates that graph classification representations, which construct local coordinate systems to extract physicochemical information interaction and residue spatial distribution from local environments, are more appropriate for representing residue local structure information. Moreover, the general outperformance of structure-based methods over sequence-based ones reaffirms the significance of protein structures in elucidating protein functions. Popular predictors like GraphBind, ScanNet and MaSIF conduct binding residue identification solely based on information interactions between close residues, information extraction from protein surfaces, or neighboring residue arrangements. The superiority of GraphRBF over them indicates that combining graph structures with point cloud structures can better characterize the latent residue binding patterns.

Table 1. Performance comparison of GraphRBF with state-of-the-art methods on protein-protein and protein-RNA/DNA binding test sets.

**Dataset**	**Method**	**Rec**	**Pre**	**F1**	**MCC**	**AUC**	**PRC**
	SPPIDER	0.436	0.192	0.271	0.199	0.726	0.179
P-250_Test	GraphPPIS	0.429	0.228	0.298	0.230	0.751	0.212
	MaSIF-site	**0.515**	0.170	0.255	0.184	0.732	0.179

	ScanNet	0.415	0.285	0.338	0.275	0.797	0.258
	GraphRBF	0.430	**0.367**	**0.396**	**0.341**	**0.825**	**0.366**
DNA-220_Test	DeepDISOBind	0.048	0.078	0.059	0.032	0.546	0.048
	DRNApred	0.237	0.118	0.158	0.123	0.699	0.096
	TargetDNA	0.461	0.187	0.266	0.251	0.824	0.176
	GraphBind	**0.608**	0.247	0.351	0.354	0.903	0.289
	GraphRBF	0.581	**0.320**	**0.412**	**0.403**	**0.920**	**0.341**
RNA-374_Test	DeepDISOBind	0.196	0.295	0.235	0.188	0.699	0.215
	DRNApred	0.103	0.206	0.137	0.096	0.539	0.127
	hybridRNAbind	0.346	0.416	0.378	0.333	0.774	0.340
	GraphBind	0.473	0.403	0.435	0.385	0.826	0.406
	GraphRBF	**0.576**	**0.476**	**0.522**	**0.480**	**0.902**	**0.512**

Comparison on individual proteins. To detail the performance of GraphRBF and other compared predictors at the level of individual proteins within the test set, we calculated the F1-scores for GraphRBF and each compared method on individual proteins in the three test sets and illustrate the differences relative to GraphRBF (see Fig. 2). The results reveal that GraphRBF has the fewest proteins with the lowest F1-scores (0-0.3) and the highest number of proteins with the highest F1-scores (0.6-1) among all compared methods.

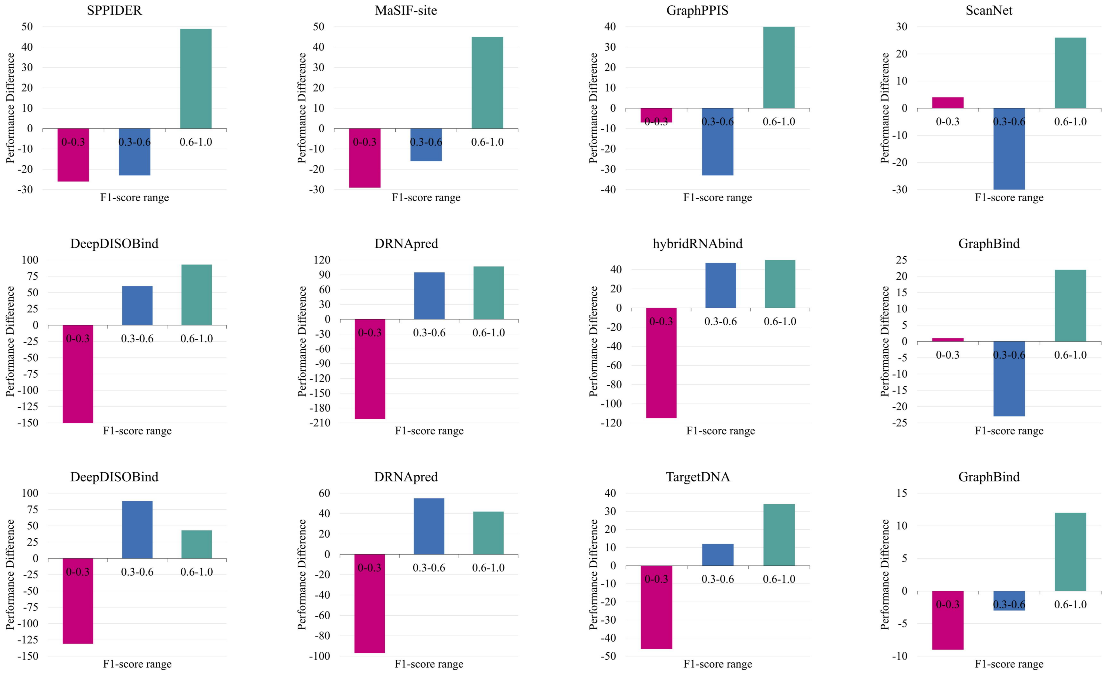

Fig. 2 Performance comparison on individual proteins. Difference in the number of proteins between the GraphRBF and the other compared methods at three F1-score distributions (0-0.3 low, 0.3-0.6 normal and 0.6-1.0 high) on P-250_Test, DNA-220_Test, RNA-374_Test, respectively.

Ablation studies on GraphRBF

In this section, we conduct multiple ablation experiments to evaluate the impact of different components and conditions on the performance of GraphRBF. We perform different settings on the test set to validate performance changes under different conditions including: hyperparameter tuning, component ablation, radial basis function ablation, and feature ablation.

Hyperparameter tuning experiments. We conduct hyperparameter tuning experiments for GraphRBF and select the best hyperparameters on the validation set. The models, trained with various configurations, were evaluated on the test set; the outcomes for protein-protein binding are presented in Table 2, while those for nucleic acid binding proteins are detailed in Supplementary Tables S1 and S2.

As shown in experiments A and B (see Table 2), smaller neighborhood radius which represents smaller local structure limit the sensory field of the network and results in poorer performance. Experiments C and D show that fewer filters and fewer kernels lead to inadequate feature extraction and thus performance degradation. Experiments E and G indicate that 2 representation layers with a hidden size of 256 is the best setting on this test set, while 1 representation layer with a hidden size of 128 shows the best performance on the nucleic acid binding protein test set. Experiment F shows that the model performs better with smaller embedding dimensions of location information. To better display the results of the ablation studies, we also illustrate the changes in F1-scores across different parameter settings for the three test sets (as shown in Fig. 3).

Table 2. Ablation studies on protein-protein binding test set P-250_Test with different settings of hyperparameters.

	r[a]	d[b]	G[c]	N_f[d]	K[e]	D_p[f]	D[g]	Rec	Pre	F1	MCC	AUC	PRC
Base	20	10	32	64	2	64	256	0.430	**0.367**	**0.396**	**0.341**	0.825	**0.366**
A	15							0.438	0.343	0.385	0.327	0.825	0.350
	25							0.481	0.329	0.390	0.334	0.823	0.347
B		5						0.460	0.342	0.392	0.335	0.829	0.354
		15						0.440	0.354	0.392	0.335	0.828	0.361
C			16					**0.487**	0.327	0.391	0.335	0.820	0.357
			64					0.426	0.354	0.386	0.329	0.822	0.356
D				32				0.479	0.312	0.378	0.320	0.821	0.345
				128				0.412	0.336	0.370	0.311	0.821	0.336
E					0			0.445	0.321	0.373	0.313	**0.830**	0.347
					1			0.418	0.366	0.390	0.334	0.828	0.355
					4			0.452	0.320	0.375	0.316	0.821	0.347
F						3		0.457	0.342	0.391	0.334	0.826	0.356
						32		0.466	0.344	0.389	0.331	0.826	0.355

	128		0.466	0.321	0.380	0.322	0.818	0.341
G		64	0.452	0.315	0.371	0.312	0.824	0.336
		128	0.406	0.363	0.383	0.327	0.818	0.348
		512	0.482	0.313	0.379	0.322	0.823	0.346

[a]Radius of the structural neighborhood: it defines the point cloud within the neighborhood of the target residue, and its unit is Å.

[b]The distance threshold to get the adjacency matrix: it defines the threshold for concatenating edges between residues, and its unit is Å.

[c]The number of GDG kernels in each filter.

[d]The number of filters used in PRBFNN.

[e]The number of representation layer. Layer 0 stands for the encoder layer.

[f]The dimension D_p of encoded position information vector using local coordinates.

[g]The dimension of encoded node, edge and graph feature vector. We set $D_e = D_v = D_u$.

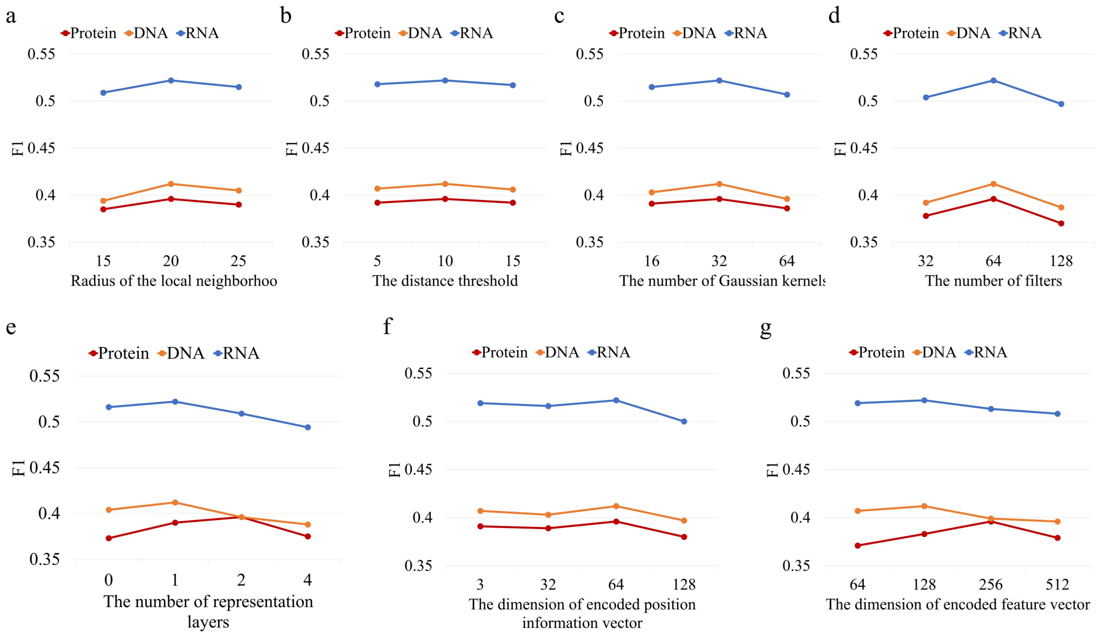

Fig. 3 Trend plots of F1-score with different parameter settings. Changes in F1-scores on the three test sets with various parameter settings: **a,** Radius of structural neighborhood, 15, 20 and 25. **b,** The distance threshold for the adjacency matrix, 5, 10 and 15. **c,** The number of GDG kernels, 16, 32 and 64. **d,** The number of filters, 32, 64 and 128. **e,** The number of representation layers, 0, 1, 2 and 4. **f,** The dimension of embedded position information vector, 3, 32, 64 and 128. **g,** The dimension of embedded feature vector, 64, 128, 256 and 512.

Component ablation experiments. Next, we conduct ablation experiments on different components of the model. We retrain the model and compare its performance when removing the EGNN, PRBFNN, and attention of kernels in PRBFNN, respectively, on the protein-binding test set, with operation details provided in Supplemental Method S2. When

using EGNN alone, GraphRBF describes graph features based only on the learned node features,omitting the spatial distribution information of the nodes, which loses a lot of important structural information. When using PRBFNN alone, GraphRBF focuses too much on the spatial distribution of nodes and almost ignores the associations between nodes as well as the information interaction within the neighborhood. When using PRBFNN without attention in combination with EGNN, GraphRBF aggregates features through direct summation rather than weighted summation using the attention coefficients calculated by fusing information about the spatial distribution of residues with its own features. This omission results in the model failing to pay attention to the kernels that are more important for information fusion and spatial distribution representation, to the extent that important real structural information is lost.

As shown in Fig. 4a, removing the attention module significantly reduces performance, which demonstrates the importance and effectiveness of the attention module in calculating the attention coefficients for feature extraction through GDG kernel functions and node features. While the use of EGNN and PRBFNN alone achieves good results, the results gain a higher level of enhancement when combining them together, which further proves the effectiveness of our hierarchical learning strategy as well as the effectiveness of combining graph analysis with point cloud analysis. The percentages of average time spent per epoch of training for the model under different component settings in terms of our current default parametric model are also shown in Fig. 4a. The component ablation experiments on protein-DNA/RNA binding sets are shown in Fig. S1a and Fig. S2a; and the specifics on the three test sets are shown in Table S3.

Radial basis function ablation experiments. We also tested the radial basis function[27,28] in the PRBFNN module by using GDG function, the original Gaussian function, inverse quadratic function, and inverse multiquadratic function, and the definitions of the above functions are described in Supplemental Method S3. As shown in Fig. 4b, Gaussian kernel is weaker than the GDG kernel and increases the memory and time for training the model. The percentages of average time spent per epoch of training for the model under different radial basis function settings in terms of our current default parametric model are also shown in Fig. 4b, which shows that the overall difference in training time consumed using different kernel functions is small. The function ablation experiments on protein-DNA/RNA binding set are shown in Fig. S1b and Fig. S2b and the specifics are also shown in Table S3.

Feature ablation experiments. Finally, we evaluate the performance of GraphRBF under different feature combinations: (i) each feature alone, (ii) evolutionary information PSSM+HMM and its combination with other features, (iii) RT combined with SS and AF, respectively, and all three combination, (vi) each of the five features removed in turn, and (v) all of them PSSM+HMM+SS+RT+AF. By sequentially removing each feature from the input

features and retraining the model with the remaining features, we observe three generally consistent F1-score trends across the three test sets, as shown in Fig. 4c.

The results indicate that using PSSM or HMM alone is more effective than other individual features, with HMM being more influential than PSSM. The combination of evolutionary information with structural features yields further enhancement. Structural information is relatively more important on the protein-binding dataset, while evolutionary information plays a more significant role on nucleic acid-binding proteins. Amino acid type features contribute positively to model enhancement, whereas atomic features exhibit average performance with minimal enhancement.

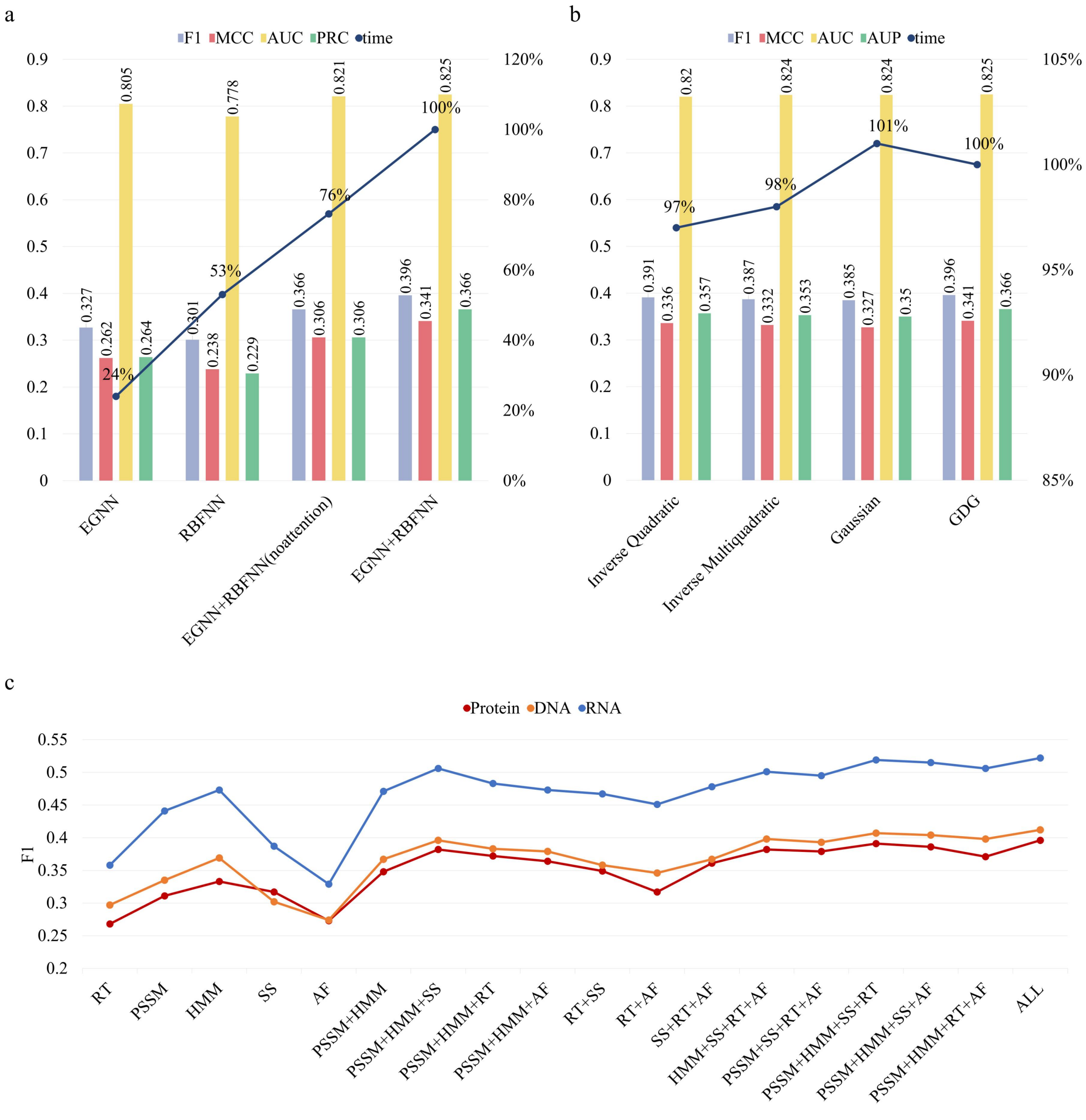

Fig. 4 Component and feature ablation experiments. **a,** Performance of GraphRBF with different combinations of components on P-250_Test. **b,** Performance of GraphRBF with different kernel functions on P-250_Test. The blue lines in (a) and (b) are the percentage line

of average time spent per epoch of training, in terms of our current default parametric model. **c,** Trend plot of F1-score with different feature combinations on the three test sets.

Interpretative analysis of the model

To gain a deeper understanding of distinct binding patterns, as well as the GDG kernels and high-level representations, we undertake data cross-training and independent testing, feature visualization, and interpretability analysis of the GDG kernels and the learned high-level representations. The parameters of each GDG kernel are selected from the 64 filters in last representation layer of the trained model.

Data cross-training. In this section, we aim to explore the relationships between datasets from different protein binding site types by performing data cross-training and independent testing. We merged the two nucleic acid-binding protein training sets to create the NA cross-training dataset and retrained our model with the previously determined optimal parameters. We then individually tested the model's performance on the DNA-220_Test and RNA-374_Test datasets.

Furthermore, we merged the NA cross-training dataset with the protein-binding protein training set to form the PA cross-training dataset, retraining our model to evaluate its performance across the DNA-220_Test, RNA-374_Test, and P-250_Test datasets. Fig. 5 presents the comparative results against separate training for each dataset. The results indicate that cross-training with NA data significantly enhances the model's performance on the DNA-220_Test and RNA-374_Test, likely due to the similarity in binding patterns and data distribution between DNA and RNA when interacting with proteins. Additionally, the inclusion of NA data broadens and diversifies the training set, bolstering the model's generalization capability and mitigating overfitting. Conversely, mixing protein-binding protein data with NA data leads to a decline in GraphRBF's performance across all three test sets, possibly because the binding patterns of protein-protein interaction sites differ from those of nucleic acid protein interaction sites[7,29].

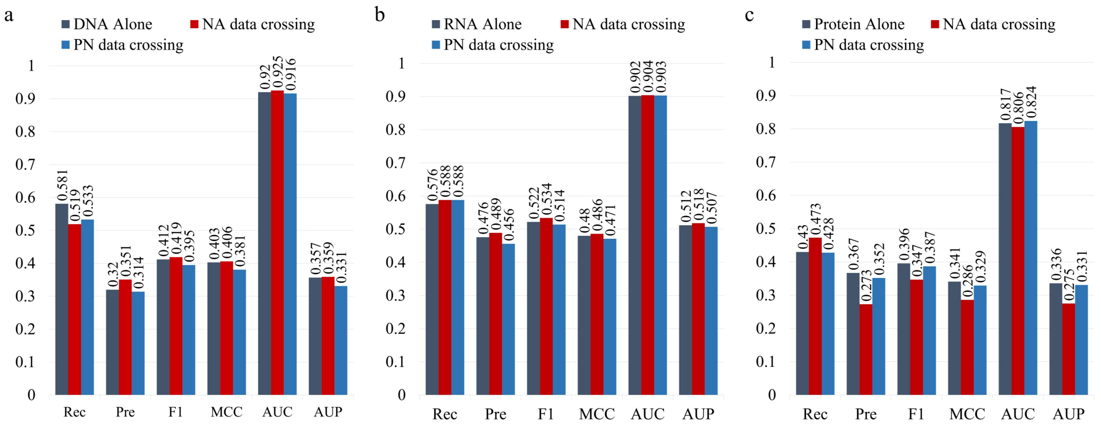

Fig. 5 Performance comparison between cross-training and training alone. Results from the three training sets of different protein binding site types, NA crossing training set, and PA crossing training set on each test set were used individually: **a,** DNA-220_Test. **b,** RNA-374_Test. **c,** P-250_Test.

Interpretation and visualization of GDG kernels. The analysis delves into the centers and widths of GDG kernels represented by FocusGauss RBF module (FGM), as shown in the Methods section. In this study, center μ and width σ of each GDG kernel in each filter serve as learnable parameters that are initialized randomly. This approach offers several benefits in terms of interpretability and model flexibility. By treating these parameters as learnable, the model learns to adapt its representation to the underlying data distribution in a data-driven manner and effectively models complex spatial distribution patterns and relationships of data.

The distribution of the learned centers across the 2,048 GDG kernels, deployed in 64 filters with a configuration of 32 kernels per filter, is depicted in Fig. 6a using Kernel Density Estimation (KDE)[30,31]. The visualization of KDE results, enhanced through coloring, improves the interpretability of density distribution patterns and facilitates the identification of variations in spatial density[32].

Additionally, examining the distribution of width, shown in Fig. 6b, provides information about the scale and spread of individual kernels. We also randomly selected four filters and visualize their GDG kernels using KDE to color the kernels (see Fig. 6c-f). Each kernel is represented as a sphere, with its center and width determining its position and size in the visualization. Filters with dense spatial distributions seem to capture important and frequently occurring features, while filters with sparse distributions may capture less common or less important features.

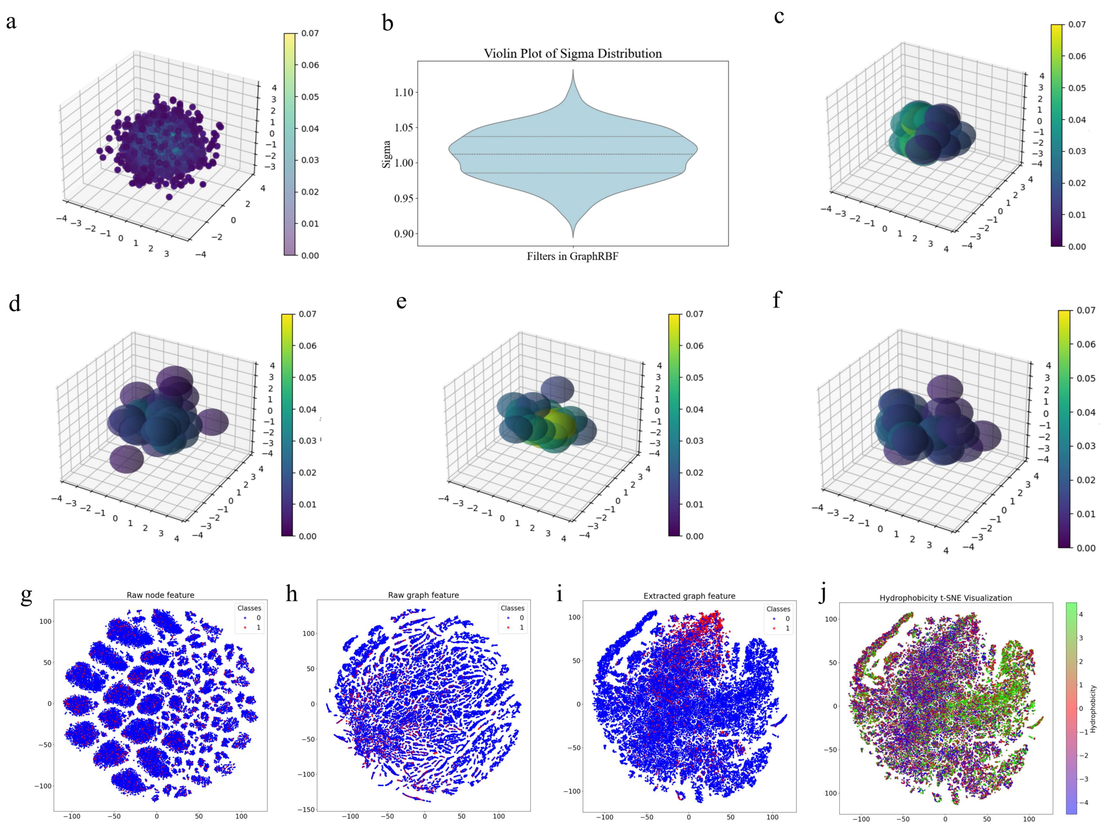

Fig. 6 Visualization of GDG kernels and learned representations. Distribution of centers (**a**) and widths (**b**) of all GDG kernels in the second representation layer learned by model trained on protein-binding protein data. **c-f,** visualization of the GDG kernels in four randomly selected filters. **g-i,** the two-dimensional projection visualization of the raw graph features, raw residue features, and extracted graph features, respectively, using t-SNE. **j,** t-SNE projection based on hydrophobicity values of amino acids.

Interpretation and visualization of learned representations. In this study, we utilized t-SNE(t-distributed Stochastic Neighbor Embedding)[33] dimensionality reduction technique to visualize the original input graph features, the raw center residue features and the learned extracted graph features derived from GraphRBF. Subsequently, we applied coloration based on binding labels, shown in Fig. 6g, 6h and 6i, and other physicochemical features for enhanced interpretation. For the target residue in P-250_Test, the sum of the raw feature vectors of all nodes within its neighborhood is used as the raw graph feature vector, with a size of 92. The latent graph feature with a size of 512 obtained by GraphRBF, is a concatenation of two embedded graph features from two representation layers, which is then fed into the classifier. We extracted the following five common physicochemical properties for each residue[34,35]: atomic number, charge, number of hydrogen bonds, side chain pKa and hydrophobicity value. In Fig. 6j, we visualize the learned graph features by colouring the residue points using their hydrophobicity values.

The t-SNE visualization in Fig. 6i, featuring the GraphRBF learned representations, shows a clear clustering of positive samples within specific areas, which not only indicates the capability of GraphRBF in distinguishing essential attributes of positive samples effectively but also highlights a refined and unified representation of key patterns in the learned feature space. In contrast, the visualizations in Fig. 6g and 6h reveal a scattered distribution of the raw graph features and residue features, indicating a lack of separation between positive and negative samples. The broader spread of original features underscores the complexity and variability present within the dataset, highlighting the challenges associated with accurately distinguishing between positive and negative samples.

In addition, the t-SNE visualization in Fig. 6j, based on the learned representations, demonstrates that dark and light colors (representing different levels of hydrophobicity) are roughly separated in the visualization chart, which suggests that GraphRBF is learning to indirectly capture and encode relevant information pertaining to hydrophobicity, a critical physicochemical property in many biological and chemical contexts. The other physicochemical features are also visualized and shown in Supplemental Fig. S3, and the interpretation and visualization results of DNA-220_Test and RNA-374_Test are shown in Fig. S4 and S5, which similarly suggest that GraphRBF seems to capture the essential properties of amino acids during its learning process.

Applying GraphRBF to exploring the SARS-CoV-2 spike protein

In this section, we predicted and visualized the binding sites of the spike glycoprotein of acute respiratory syndrome coronavirus 2, SARS-CoV-2 spike, by GraphRBF. The SARS-CoV-2 spike protein is essential for the virus's ability to bind to and enter host cells by attaching to the human ACE2 receptor[36,37]. Understanding this interaction is critical for deciphering how the virus infects human cells[36]. The SARS-CoV-2 spike protein is a homotrimer, meaning it is composed of three identical polypeptide chains A, B, and C[36]. Each chain contributes to the formation of the trimeric spike protein structure.

We use the spike structure 7TGW[38] from the omicron variant of SARS-CoV-2 to predict its protein binding sites, especially BCEs, which are defined as residues directly involved in a antibody–antigen complex. We show the scores of each residue on chain A of the spike protein in Fig. 7a, predicted by GraphRBF trained on protein-binding protein set. We also categorize the 11 predicted regions (labeled from left to right) by sequence and structural nearest neighbor relationships (Fig. 7a). The NTD (N-terminal Domain) is a flexible region of the spike protein and contains multiple epitopes that are crucial for antibody binding[36,39]. The prediction results indicate high scores for the NTD region, suggesting that GraphRBF correctly identifies antibody binding sites within this area[36,39]. The RBD (Receptor Binding Domain) is known to be the primary site on the spike protein that binds to the ACE2

receptor[36] of host cells . High scores in the RBD are expected since this region is a major target for neutralizing antibodies[40,41]. The high scores of RBD predicted by GraphRBF align with extensive research showing that it induces potent neutralizing antibodies and is a key area for vaccine development[36]. SD1 (Sub-domain 1) and SD2 (Sub-domain 2), being part of the S1 sub-unit, play important roles in the overall antigenicity and immunogenicity of the spike protein[41]. We observe that GraphRBF assigns high scores to them, again consistent with previous research results that they also contain antibody binding sites[36,38].

In this section, we summarize the predicted highly scored regions (i.e. regions 1, 2, 3, 4, 5, 7, 8, 11) that can be well matched to experimentally verified complex binding sites. Considering that the spike protein undergoes an actual conformational change upon binding to the antibody[36], in order to show more clearly the predicted results with respect to experimental resolved antigen-antibody binding situations, we mapped the predicted residue scores of the unbound spike proteins to the corresponding residues in the complexes. We find that GraphRBF successfully predicts binding sites to their BCEs, especially to nanobodies, just from the unbound spike protein structure. Nanobodies are the smallest known single-domain antibodies with therapeutic potentials and show great translational potential in preclinical and clinical studies[42,43]. In recent years, some anti-SARS-CoV-2 nanobodies, named Nanosota, have been discovered from an immunized alpaca[44]. We show the experimental solved results based on the latest update (2024-02-21) of the molecular model of the SARS-CoV-2 spike/nanobody mixture complex 8G70[45] and the predicted binding interface of GraphRBF in Fig. 7b. It can be seen that the continuous binding regions predicted by GraphRBF almost coincides with those of the experimental solved complexes. Through Fig. 7b and prediction regions, we can see that prediction region 1 corresponds to the Nanosota-5 binding region, region 5 to Nanosota-3, region 7 to Nanosota-6, and region 8 to Nanosota-5 and 6. Simultaneously, to further explore the binding situation of the predicted region 1 (Fig. 7b) with Nanosota-3, we enlarge the binding interface in Fig. 7c and find that all the binding amino acids are correctly identified.

We also find that GraphRBF predicts some residue sites that bind to other proteins. We show these sites in the predicted mapping result plots for complex 7L2C[46] (Fig. 7e) with neutralizing antibody 2-51 binding NTD and complex 7YH6[47] (Fig. 7f) with neutralizing antibody NIV-8 binding RBD. GraphRBF assigns high scores for all the binding regions of antibodies 2-51 (predicted region 3) and NIV-8 (predicted region 4 and 5). A common sugar-free, positively charged surface (defined by the sugar moieties of N17, N74, N122, and N149) is also identified by GraphRBF (predicted region 1 and 2), which is one of the seven potent neutralizing antibodies, a predominantly targeted region also known as the ‘supersite’. It is reported that all potent SARS-CoV-2 neutralizing antibodies against NTDs may target this supersite[46]. There are also a number of reported and potentially significant sites above the

RBD[47] as follows. Y489 is identified as a viral vulnerability site and is common to broadly neutralizing antibodies against the Omicron subvariant. F486 is also a binding site for a number of antibodies, and its benzene ring may be involved in hydrophobic interactions and its position may have an effect on electrostatic interactions. The G485 mutation has a significant effect on the binding of the NIV-10 antibody binding, suggesting that G485 may be involved in critical conformational stability in antibody binding, and that its B-factor may be indicative of the flexibility or dynamics of the site. N487 is mentioned in the analysis of escape mutations in NIV-10 antibodies and may be involved in interactions with the antibody, possibly through the formation of a hydrogen bond that may be involved in binding. In addition, there are a number of highly-scored residues that are binding sites for spike proteins to the receptor ACE2[36,38], such as Y453 and Y505, which are involved in hydrophobic interactions and π-π stacking; R457, which is involved in charge interactions with the ACE2 receptor; N501, which forms a critical hydrogen bond in the RBD for ACE2 receptor binding; and K417, in the 'upper' region of the RBD, which is required for binding to ACE2 and helps to stabilize the 'upward' conformation of the RBD[48]. All these important binding sites are predicted successfully in prediction region 5. And the region 11 is located in the vicinity of Heptad Repeat 2 (HR2) and Central Helix (CH) in the S2 subunit, which are closely related to the viral membrane fusion mechanism[48,49].

Then, we also find some predicted sites that are potential binding regions for new nanobodies or even new drugs. These amino acids, T470-F497 (part of predicted region 5) in the RBD regions of all three chains of the spike protein are predicted to have high binding scores, but only the portion on chains A and B show binding to Nanosota-3 in the 8G70 complex. Through structural and sequence analyses, we find that the residue spatial distributions of the predicted region, the hydrophobic values, and the electrostatic distributions of the region on chain C are almost identical with the corresponding regions on chains A and B (see Fig. 7b and d), based on which we infer that this part of the chain C has great potential for binding to nanobodies. Also based on the reported results[49] of antibody responses in patients' sera using VirScan technology, we know that regions V551-A570 (part of predicted region 5) and A766-Q785 (region 9) were not picked up by the HMM classifier because of their short lengths; however, these regions score in multiple samples and correspond to accessible regions in the crystal structure, which suggests that they may represent true epitopes[49,50]. These regions may contain key epitopes capable of eliciting host immune responses and are likely to be potential targets in vaccine design.

Finally, there are still two predicted regions (regions 6 and 10) for which we have not yet collected information to prove whether they are binding regions or not. Considering that region 6 is located in the RBD region[37,40,50], it is also very likely that additional relevant binding site information may be identified in future studies. And region 10 is close to the S2

region related to immune escape and transmembrane action, which requires further experimental verification to determine its function[39,49].

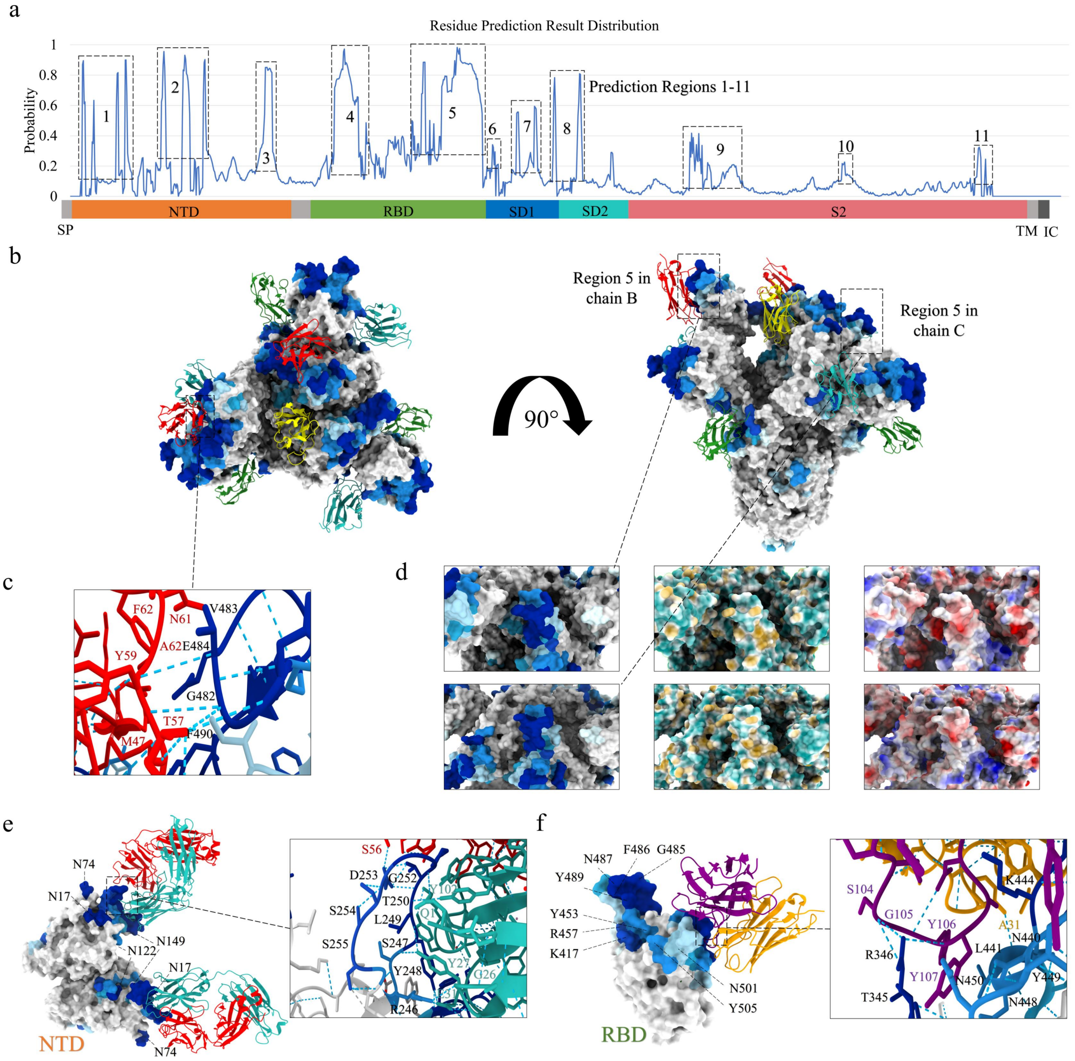

Fig. 7 The prediction results and visualizations of binding sites on the SARS-CoV-2 spike protein. a, The predicted scores of residues on A-chain of protein 7TGW. **b,** The binding sites of SARS-CoV-2 spike to nanobody predicted by GraphRBF on the surface of the protein. **c,** Distribution of the predicted scores (left), hydrophobic values (center), and electrostatic values (right) for Region 1 (top) and Region 2 (bottom). **d,** The details of binding interface prediction between spike protein and Nanosota-3. **e,f,** The details of binding prediction of NTD and antibody 2-51 complex and RBD and NIV-8 complex. The molecular surface of the query protein is shown with coloring based on predicted scores, ranging from low (white) to high (dark blue). Representative nanobodies binding the main epitopes are superimposed in color, cartoon representation. Red, yellow, green and blue cartoons represent Nanosota-3, Nanosota-4, Nanosota-5 and Nanosota-6[44], respectively. The visualization software used in this study is ChimeraX[51].

Discussion

The development of accurate and interpretable models for predicting protein-protein and protein-nucleic acid interaction sites is crucial for advancing our understanding of biological processes and facilitating disease diagnosis and drug design. The local structural context of a protein is pivotal in determining the propensity of its constituent residues to engage in interactions with other biomolecules. However, fully uncovering these structural patterns remains a challenge with traditional feature extraction methods and single molecular representation of proteins. The newly designed GraphRBF is an end-to-end interpretable hierarchical geometric deep learning model that leverages deep residue information interaction based on molecular graphs and carefully represents residue spatial distributions based on residue point clouds for addressing this challenging task. GraphRBF is trained on datasets for both protein-protein and protein-nucleic acid binding sites and achieves superior performance compared to other state-of-the-art models.

The contributions and innovations of GraphRBF can be attributed as follows. (i) It extracts residue neighborhoods with local scales to obtain a stable representation of the local spatial structure of a protein, which facilitates it in capturing the true local spatial distribution of residues with respect to residue binding patterns. (ii) It combines graph structure and point cloud structure for extracting hierarchical protein molecular structure representations. The enhanced graph neural network is able to learn physicochemical information interactions of neighboring residues and the prioritized radial basis function neural network is capable of capturing the local residue spatial distributions. (iii) We applied GraphRBF on the prediction and visualization of the spike glycoprotein of SARS-CoV-2 omicron variant. GraphRBF not only successfully predicts all the known binding regions and important binding sites with different antibodies on the NTD, RBD, SD1, and SD2 regions, but also identifies multiple potential antibody binding regions and receptor binding sites with biological evidence.

Despite the success of GraphRBF in capturing essential features of protein function sites, there remains room for improvement in extracting spatial distribution information comprehensively. Current methodologies may not fully leverage the intricate spatial arrangements of amino acids within protein structures, potentially limiting their predictive capabilities. At the same time, the current version of GraphRBF only utilizes protein local structures, ignoring information of the overall structure, which may also limit its performance. In the future, new models for more comprehensive extraction of residue spatial distributions combined with physicochemical information interaction that is conducted on the entire protein structures have the potential to achieve better performance.

In conclusion, GraphRBF is a hierarchical and interpretable geometric deep learning framework for learning residue binding patterns. While it represents a great step forward in predicting protein interactions, there are still challenges to overcome and opportunities to

explore. We believe that GraphRBF will contribute to the function and mechanism analyses of new proteins as well as technical guidance for disease diagnosis and drug design.

Methods

Dataset Curation and Feature Preparation

In this section, we introduce the protein binding site data as well as the feature preparation that includes three parts: extraction of node features, extraction of residue neighborhood and construction of local coordinate system, and construction of local graphs and extraction of edge features.

Dataset curation. In this study, we train and test GraphRBF on protein-protein and protein-nucleic acid interaction site datasets separately and compare them with other leading methods. In order to compare it with other methods, the protein-protein interaction datasets were obtained from Protein-Protein Docking Benchmark 5.5[52] (DBD 5.5) and Dockground[53], and the nucleic acid binding proteins were collected from the BioLiP2[54] database. The detailed processing of the datasets (e.g., sample selection based on resolutions, removal of redundant proteins according to sequence similarity, and separation of training and test sets) is shown in Supplemental Method S4. Eventually, we obtain 1001 protein-binding proteins, 881 DNA-binding proteins, and 1497 RNA-binding proteins as the training sets; 250 protein-binding proteins, 220 DNA-binding proteins, and 374 RNA-binding proteins as the test sets. The details of the datasets are summarized in Supplemental Table S4.

Extraction of node features. Multiple types of amino acid features are extracted that include atomic features of residues, secondary structure encoding, evolutionary information, and residue type encoding. Seven atomic features are extracted for each heavy atom belonging to the target residue based on the PDB file: atom mass, B-factor, whether it is a residue side-chain atom, electronic charge, the number of hydrogen atoms bonded to it, whether it is in a ring, and the van der Waals radius of the atom. The DSSP[55] tool is applied to generate secondary structure features of residues including a residue water-exposed surface, five bond and torsion angles, and eight one-hot encoded secondary structure with eight states. For the evolutionary information, PSI-BLAST[56] is used to search the Uniref 90 database[57] for homologous sequences with three iterations and $e\text{-}value<10^{-3}$ to generate the position-specific scoring matrix (PSSM) with 20 dimensions. HHblits[58] is implemented to search the uniclust30 database[59] to generate an HMM matrix of the query sequence with 30 dimensions. For the residue type encoding, the 20 types of amino acids are ordered as ACDEFGHIKLMNPQRSTVWY, and an 20-dimension one-hot coding of each residue is generated accordingly. In conclusion, a node feature matrix of size $L\times91$ is constructed for each protein, in which L is the length of the protein. In addition, the original features are min-max normalized before training (see details in Supplemental Method S5).

Extraction of residue neighborhood and construction of local coordinate system. In this study, the α-carbon coordinates of each residue are used as its pseudo position. Then for each residue, its local neighborhood is extracted as a sphere with a radius r centered on it. In order to ensure the rotation and translation invariance of the protein local structure, we construct a local coordinate system for the neighborhood of residue a as follows. The coordinate origin is selected as residue a and the first direction vector f_1 is built based on residue a and its closest neighboring residue b by the following formula.

$$f_1 = \frac{x_b - x_a}{||x_b - x_a||}$$

where x_a and x_b represent the coordinates of residue a and b in the original pdb file. Then, the second direction vector f_2 can be generated based on residue a and the centroid of its side chain as follows.

$$f_2 = \frac{x_s - x_a}{||x_s - x_a||}$$

where x_s represent the coordinates of the centroid of the side chain of residue a. The third and fourth direction vectors f_3 and f_4 are then constructed by cross-producting f_1 with f_2, and f_1 with f_3 as follows.

$$f_3 = \frac{f_1 \times f_2}{||f_1 \times f_2||}$$

$$f_4 = \frac{f_1 \times f_3}{||f_1 \times f_3||}$$

Finally, the x, y, and z axes of the local coordinate system are respectively defined as the direction vectors f_4, f_2, and f_3 and each residue belonging to the neighborhood receives a new positional coordinate p_l in the local coordinate system.

Construction of local graphs and extraction of edge features. In this study, a local graph is defined as $G = (V, C, A, E, U)$. $V = \{v_i\}$ and $v_i \in \mathbb{R}^{D_v}$ denote the node feature vector set and the feature vector of residue i, respectively. And then, a structure-based positional embedding PE_i of residue i is introduced as the 92-*th* node feature as follows.

$$PE_i = \frac{1}{r}||p_i - p_0||$$

where p_0 and p_i denote the local coordinates of the target residue a and node i. $C = \{p_i\}$ and $p_i \in \mathbb{R}^3$ respectively denote the set of node local coordinates and the local coordinates of node i. A is the adjacency matrix of the nodes in the graph with $A_{ij} = 1$ if the spatial distance d_{ij} between node i and node j is no more than d (d is a hyperparameter and set to be 20 in this study). $E = \{e_{ij}|A_{ij} = 1\}$ denotes the edge feature vector set, where e_{ij} denotes the feature vector of the edge $i \rightarrow j$. We extracted four features for each edge $i \rightarrow j$: (1) the edge positional vector $\vec{e}_{ij}$, (2) the spatial distance d_{ij} of nodes i and j, (3) the cosine of the angle θ_{ij} between

the direction vectors of the two endpoints, and (4) the local coordinates of the two endpoints. The detailed definition of the four features are as follows.

$$\vec{e}_{ij} = p_i - p_j$$

$$d_{ij} = \|p_i - p_j\|$$

$$\cos(\theta_{ij}) = \frac{p_i \cdot p_j}{\|p_i\|\|p_j\|}$$

where p_i and p_j denote the local coordinates of the two endpoints. Finally, we concatenate these four features to get an edge feature vector $e_{ij} \in \mathbb{R}^{11}$. $U = \{u_i\}$ denotes the graph feature vector set to be calculated, which will be described in the next section.

The GraphRBF model

The following section describes the pipeline of the edge-node-pos-graph model (ENPGM) encompassing an enhanced graph neural network, a prioritized radial basis function neural network, and a calculation of residue binding probability (see Fig. 8 for the neural network architecture of GraphRBF). Here, $v \in \mathbb{R}^n$ denotes a vector of 1 column and n rows.

Enhanced graph neural network. We design an enhanced graph neural network (EGNN) incorporating residue position encoding and feature updating according to the local graph structure. This module contains two main parts: the encoding layer and the representation layer.

At the encoding layer (see Fig. 8), the node feature vector set V, the edge feature vector E, and the local coordinate set C serve as the input to EGNN. After entering EGNN, each edge feature vector e_{ij} and two node feature vectors v_i and v_j of its two endpoints are first concatenated and the edge feature vector $e_{ij}^0 \in \mathbb{R}^{D_e}$ is then encoded as follows.

$$e_{ij}^0 = MLP_e^0([v_i\,;\, v_j\,;\, e_{ij}])$$

where $[\,;\ ;\,]$ stands for the concatenation operation. Then, the node feature vector $v_i^0 \in \mathbb{R}^{D_v}$ is encoded from v_i, the original local coordinates p_i, and the sum aggregation of encoded edge feature vectors e_{ij}^0 as follows.

$$v_i^0 = MLP_v^0\left(\left[v_i\,;\, p_i\,;\, \sum_{j \in N(v_i)} e_{ij}^0\right]\right)$$

where $N(v_i)$ is the set of neighbors of node i. Finally, the position information feature vector $p_i^0 \in \mathbb{R}^{D_p}$ is updated by only encoding the original local coordinates p_i as follows.

$$p_i^0 = MLP_p^0(p_i)$$

At the k-th representation layer (where $k = 1,2,\ldots,K$) (see Fig. 8), the features of edges, nodes, positions, and graphs are updated. First of all, each edge feature vector is updated as follows.

$$e_{ij}^k = e_{ij}^{k-1} + \tilde{e}_{ij}^k$$

$$\tilde{e}_{ij}^k = MLP_e^k\left(\left[v_i^{k-1};\ v_j^{k-1};\ e_{ij}^{k-1}; p_i^{k-1}; p_j^{k-1};\ \vec{e}_{ij}^{\,k};u^{k-1}\right]\right)$$

$$\vec{e}_{ij}^{\,k} = p_j^{k-1} - p_i^{k-1}$$

where u^{k-1} is the graph feature vector of layer k-1 generated by the FGM module to be explained in the next section. Then, each node feature vector is updated based on the updated edge features by the following formulas.

$$v_i^k = v_i^{k-1} + \tilde{v}_i^k$$

$$\tilde{v}_i^k = MLP_v^k\left(\left[v_i^{k-1};\ p_i^{k-1};\ \sum_{j\in N(v_i)} e_{ij}^k\ ;\ u^{k-1}\right]\right)$$

And the position feature vector can be updated as follows.

$$p_i^k = p_i^{k-1} + \tilde{p}_i^k$$

$$\tilde{p}_i^k = MLP_p^k\left(\left[p_i^{k-1};\ p_i\right]\right)$$

In this study, the above MLP operations are a point-by-point nonlinear transformation module consisting of two linear layers, a Batch Normalization layer[60], a rectified linear unit (ReLU) layer[61], and a Dropout layer[62] as follows.

$$MLP(X) = W_2\left(\text{Dropout}\left(\text{ReLU}\left(\text{BN}(W_1X + b_1)\right)\right)\right) + b_2$$

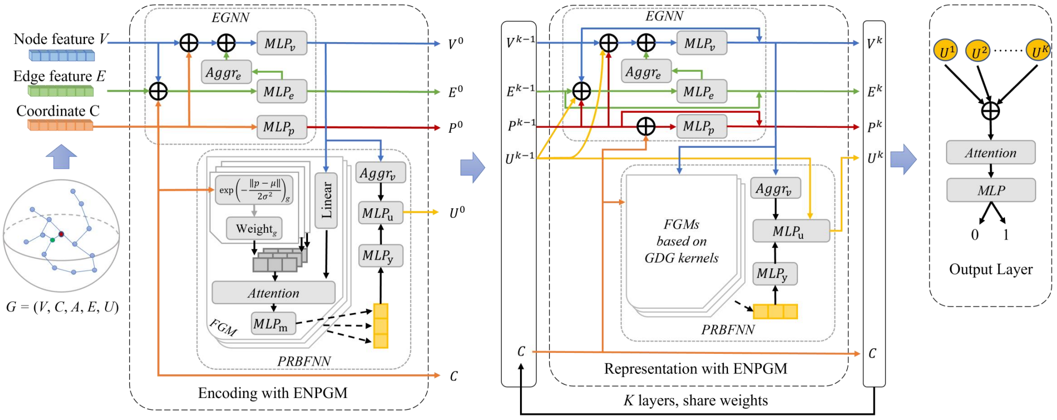

Fig. 8 The diagram of GraphRBF network. The network contains an ENPGM-based feature encoding layer, multiple ENPGM-based representation layers, and an output layer containing an attention mechanism and an MLP. The encoding layer encodes the input node and edge features into a high-level representation of fixed dimensions, encodes the local coordinates of the residues as location-informative features, and also computes graph features based on the local coordinates and the updated node features. The K representation layers update the feature representations of edges, nodes, and locations through the graph structure and updates the graph feature vectors using the radial basis module. Finally, the graph features obtained from each representation layer are concatenated and input to the output layer for prediction and classification of binding residues.

Prioritized radial basis function neural network. In the module, graph features are encoded based on the local spatial distribution combined with the encoded node features of the residues in the neighborhood by using a set of trainable spatial convolution filters and an attention mechanism[63], which we refer to as the FocusGauss RBF module (FGM). The FGM module also contains an encoding layer and a representation layer.

At the encoding layer, the FGM takes as inputs a set of nodes with original local coordinates and original node feature vectors and outputs a N_f-dimensional feature $y^0 = \left(y_1^0, y_2^0, \dots, y_{N_f}^0\right)^T$ (N_f represents the number of filters and is a adjustable hyperparameter), where the element y_m^0 in the m-th dimension represents the output of the m-th filter. Each filter is parameterized using G kernels and a kernel attention (G represents the number of GDG kernels and is also an adjustable hyperparameter). Specifically, we first use a linear layer $\left(\check{v}^0 = W^0 v^0 + b^0\right)$ to transform each node feature vector into a D_v' dimensional vector, and then use the outputs of each GDG kernel function as well as the attention coefficients to perform node and GDG kernel aggregation, respectively, and finally, we use another MLP to condense the aggregated feature into the output of this filter. The process of the FGM module can be summarized as follows.

$$y_m^0 = MLP_m^0\left\{\sum_{g=1}^{G} Att_g^0\left[\sum_{i=1}^{N_{residues}} g_g^0\left(\mu_g^0, \sigma_g^0, p_i\right)\check{v}_i^0\right]\right\}$$

$$MLP_m^0(X) = BN\left(W_2^0\left(\mathrm{Dropout}\left(\mathrm{ReLU}\left(\mathrm{BN}\left(W_1^0 X + b_1^0\right)\right)\right)\right) + b_2^0\right)$$

$$Att^0 = \mathrm{softmax}\left(Q^0 O^0\right)$$

$$O^0 = \sum_{i=1}^{N_{residues}} \left(\check{v}_i^0 \otimes \tilde{g}_i^0\right)$$

For the above formulas, $W_1^0 \in \mathbb{R}^{D_v \times D_v'}$ and $W_2^0 \in \mathbb{R}^{1 \times D_v}$ in the MLP_m^0 are two trainable weight tensors and $b_1^0 \in \mathbb{R}^{D_v}$ and $b_2^0 \in \mathbb{R}^1$ are two trainable bias terms. $g_g^0\left(\mu_g^0, \sigma_g^0, p_i\right) = \exp\left(-\frac{\|p_i - \mu_g^0\|}{2{\sigma_g^0}^2}\right)$ denotes the output of node i by the g-th GDG kernel with center μ_g^0 and variance σ_g^0. $\otimes$ denotes the outer product. $\tilde{g}_i^0 = \left(g_{1i}^0, g_{2i}^0, \dots, g_{Gi}^0\right)$ is the GDG kernel output vector of node i. And the element O_{jg}^0 of matrix $O^0 \in \mathbb{R}^{D_v' \times G}$ can be expressed as $O_{jg}^0 = \sum_{i=1}^{N_{residues}} g_g^0\left(\mu_g^0, \sigma_g^0, p_i\right)\check{v}_{ij}^0$, where $\check{v}_{ij}^0$ is the j-th element of $\check{v}_i^0$. $Att^0 \in \mathbb{R}^{1 \times G}$ denotes the attention weight of the G GDG kernels calculated by using the softmax function based on a learnable vector $Q^0 \in \mathbb{R}^{1 \times D_v'}$ and O^0, and Att_g^0 d enotes the g-th element of Att^0, which is also the attention weight of the g-th GDG kernel. The feature extraction using multiple filters

mentioned above has several advantages over the general multilayer perceptron[22,64,65] and spherical harmonic function[66], which is described in Supplemental Method S6.

Later we use an MLP to transform y^0 into an intermediate vector $\breve{u}^0 \in \mathbb{R}^{D_u}$ followed by another embedding via concatenating the aggregation of the updated node feature vectors to generated the graph feature vector $u^0 \in \mathbb{R}^{D_u}$ as follows.

$$u^0 = MLP_u^0\left(\left[\breve{u}^0\ ;\ \sum_{i=1}^{N_{residues}} v_i^0\right]\right)$$

$$MLP(X) = W_2\left(\mathrm{Dropout}\left(\mathrm{ReLU}\left(\mathrm{BN}(W_1X + b_1)\right)\right)\right) + b_2$$

$$\breve{u}^0 = MLP_y^0(y^0)$$

At the k-th representation layer (where k = 1,2,…,K), the inputs are the updated node feature vectors, the original local coordinates, and the graph feature vector generated from the last layer. The definition of each filter in representation layer k is the same as that of the encoding layer as follows.

$$y_m^k = MLP_m^k\left\{\sum_{g=1}^{G} Att_g^k\left[\sum_{i=1}^{N_{residues}} \mathcal{g}_{gi}^k \breve{v}_i^k\right]\right\}$$

$$MLP_m^k(X) = BN\left(W_2^k\left(\mathrm{Dropout}\left(\mathrm{ReLU}\left(\mathrm{BN}(W_1^kX + b_1^k)\right)\right)\right) + b_2^k\right)$$

$$Att^k = \mathrm{softmax}(Q^kO^k)$$

$$O^k = \sum_{i=1}^{N_{residues}} (\breve{v}_i^k \otimes \tilde{\mathcal{g}}_i^k)$$

$$\breve{v}^k = W^kv^k + b^k$$

Different from the graph feature extraction at the encoding layer, after applying the MLP_y^k to transform $y^k = \left(y_1^k, y_2^k, \ldots, y_{N_{filter}}^k\right)^T$ into $\breve{u}^k \in \mathbb{R}^{D_u}$, we add a concatenation of the graph feature vector u^{k-1} of the last layer and a residual connection to generate the graph feature vector u^k as follows.

$$u^k = u^{k-1} + \tilde{u}^k$$

$$\tilde{u}^k = MLP_u^k\left(\left[u^{k-1}\ ;\ \breve{u}^k\ ;\ \sum_{i=1}^{N_{residues}} v_i^k\right]\right)$$

$$MLP(X) = W_2\left(\mathrm{Dropout}\left(\mathrm{ReLU}\left(\mathrm{BN}(W_1X + b_1)\right)\right)\right) + b_2$$

$$\breve{u}^k = MLP_y^k(y^k)$$

Calculation of residue binding probability. After obtaining K graph feature vectors for K representation layers, we concatenate these feature vectors and assign a weight to each

dimension of the concatenated graph feature vector, which is then fed into a multilayer perceptron classifier to get the residue binding probability $\hat{y}$ as follows.

$$\hat{y} = \text{softmax}\left(W_2\left(\text{Dropout}\left(\text{ReLU}\left(\text{BN}(W_1\tilde{u} + b_1)\right)\right)\right) + b_2\right)$$

$$\tilde{u} = Att_u \odot [u^1; u^2; \ldots; u^K]$$

$$Att_u = \text{softmax}\left(Q_u[u^1; u^2; \ldots; u^K]\right)$$

where $Q_u \in \mathbb{R}^{KD_u \times KD_u}$ is a trainable weight matrix; $Att_u \in \mathbb{R}^{KD_u}$ is the attention weight vector of the concatenated graph feature vector $[u^1; u^2; \ldots; u^K]$; $W_1 \in \mathbb{R}^{256 \times KD_u}$; $b_1 \in \mathbb{R}^{256}$; $W_2 \in \mathbb{R}^{2 \times 256}$ and $b_2 \in \mathbb{R}^2$.

Considering that there is a serious imbalance of positive and negative samples in the binding site data, we use Focal Loss[67], which addresses this class imbalance by reshaping the standard cross entropy loss such that it down-weights the loss assigned to well-classified examples (see details in Supplemental Method S7).

Data and code availability

All data resources used are freely available from the following databases: BioLiP (https://zhanggroup.org/BioLiP), DBD5.5 (https://zlab.umassmed.edu/benchmark), Dockground (http://dockground.compbio.ku.edu/). The processed data used for training and testing the models in this study can be available at https://zenodo.org/records/10826801. The source code files for reproducing and evaluating GraphRBF is available at the GitHub repository https://github.com/Wssduer/GraphRBF.

Acknowledgements

This work was supported by the National Key R&D Program of China with code 2020YFA0712400, and the National Natural Science Foundation of China with code 62272268. The funders had no role in study design, data collection and analysis, decision to publish, or preparation of the manuscript.

Author contributions

Conceived and designed the experiments: JL. Performed the experiments: SZ JH. Analyzed the data: SZ JH JL. Contributed reagents/materials/analysis tools: SZ JL. Wrote the paper: SZ JH JL. Designed the software used in analysis: SZ. Oversaw the project: JL.

Competing interests

The authors declare that they have no competing interests.

References

1 Hartwell, L. H., Hopfield, J. J., Leibler, S. & Murray, A. W. From molecular to modular cell biology. Nature 402, C47-52, doi:10.1038/35011540 (1999).

2 Charoensawan, V., Wilson, D. & Teichmann, S. A. Genomic repertoires of DNA-binding transcription factors across the tree of life. Nucleic Acids Res 38, 7364-7377, doi:10.1093/nar/gkq617 (2010).

3 Hirota, K. et al. Stepwise chromatin remodelling by a cascade of transcription initiation of non-coding RNAs. Nature 456, 130-134, doi:10.1038/nature07348 (2008).

4 Kuzmanov, U. & Emili, A. Protein-protein interaction networks: probing disease mechanisms using model systems. Genome Med 5, 37, doi:10.1186/gm441 (2013).

5 Wells, J. A. & McClendon, C. L. Reaching for high-hanging fruit in drug discovery at protein-protein interfaces. Nature 450, 1001-1009, doi:10.1038/nature06526 (2007).

6 Yan, J. & Kurgan, L. DRNApred, fast sequence-based method that accurately predicts and discriminates DNA- and RNA-binding residues. Nucleic Acids Res 45, e84, doi:10.1093/nar/gkx059 (2017).

7 Esmaielbeiki, R., Krawczyk, K., Knapp, B., Nebel, J. C. & Deane, C. M. Progress and challenges in predicting protein interfaces. Brief Bioinform 17, 117-131, doi:10.1093/bib/bbv027 (2016).

8 Zhang, J. & Kurgan, L. SCRIBER: accurate and partner type-specific prediction of protein-binding residues from proteins sequences. Bioinformatics 35, i343-i353, doi:10.1093/bioinformatics/btz324 (2019).

9 Hu, J. et al. Predicting Protein-DNA Binding Residues by Weightedly Combining Sequence-Based Features and Boosting Multiple SVMs. IEEE/ACM Trans Comput Biol Bioinform 14, 1389-1398, doi:10.1109/tcbb.2016.2616469 (2017).

10 Zhang, F., Li, M., Zhang, J. & Kurgan, L. HybridRNAbind: prediction of RNA interacting residues across structure-annotated and disorder-annotated proteins. Nucleic Acids Research 51, e25 - e25 (2023).

11 Chen, J., Xie, Z. R. & Wu, Y. Understand protein functions by comparing the similarity of local structural environments. Biochim Biophys Acta Proteins Proteom 1865, 142-152, doi:10.1016/j.bbapap.2016.11.008 (2017).

12 Xia, Y., Xia, C. Q., Pan, X. & Shen, H. B. GraphBind: protein structural context embedded rules learned by hierarchical graph neural networks for recognizing nucleic-acid-binding residues. Nucleic Acids Res 49, e51, doi:10.1093/nar/gkab044 (2021).

13 Tubiana, J., Schneidman-Duhovny, D. & Wolfson, H. J. ScanNet: an interpretable geometric deep learning model for structure-based protein binding site prediction. Nat Methods 19, 730-739, doi:10.1038/s41592-022-01490-7 (2022).

14 Gainza, P. et al. Deciphering interaction fingerprints from protein molecular surfaces using geometric deep learning. Nat Methods 17, 184-192, doi:10.1038/s41592-019-0666-6 (2020).

15 Zeng, M. et al. Protein-protein interaction site prediction through combining local and global features with deep neural networks. Bioinformatics 36, 1114-1120, doi:10.1093/bioinformatics/btz699 (2020).
16 Stebliankin, V. et al. Evaluating protein binding interfaces with transformer networks. Nature Machine Intelligence 5, 1042-1053, doi:10.1038/s42256-023-00715-4 (2023).
17 Wu, H. et al. Spatom: a graph neural network for structure-based protein-protein interaction site prediction. Brief Bioinform 24, doi:10.1093/bib/bbad345 (2023).
18 Oldfield, T. J. Data mining the protein data bank: residue interactions. Proteins 49, 510-528, doi:10.1002/prot.10221 (2002).
19 Baldassarre, F., Menéndez Hurtado, D., Elofsson, A. & Azizpour, H. GraphQA: protein model quality assessment using graph convolutional networks. Bioinformatics 37, 360-366, doi:10.1093/bioinformatics/btaa714 (2021).
20 Wang, Y., Wu, S., Duan, Y. & Huang, Y. A point cloud-based deep learning strategy for protein-ligand binding affinity prediction. Brief Bioinform 23, doi:10.1093/bib/bbab474 (2022).
21 DeFever, R. S., Targonski, C., Hall, S. W., Smith, M. C. & Sarupria, S. A generalized deep learning approach for local structure identification in molecular simulations. Chem Sci 10, 7503-7515, doi:10.1039/c9sc02097g (2019).
22 Qi, C., Su, H., Mo, K. & Guibas, L. J. PointNet: Deep Learning on Point Sets for 3D Classification and Segmentation. 2017 IEEE Conference on Computer Vision and Pattern Recognition (CVPR), 77-85 (2016).
23 Li, P. & Liu, Z. P. GeoBind: segmentation of nucleic acid binding interface on protein surface with geometric deep learning. Nucleic Acids Res 51, e60, doi:10.1093/nar/gkad288 (2023).
24 Porollo, A. & Meller, J. Prediction-based fingerprints of protein-protein interactions. Proteins 66, 630-645, doi:10.1002/prot.21248 (2007).
25 Yuan, Q., Chen, J., Zhao, H., Zhou, Y. & Yang, Y. Structure-aware protein-protein interaction site prediction using deep graph convolutional network. Bioinformatics (2021).
26 Zhang, F., Zhao, B., Shi, W., Li, M. & Kurgan, L. DeepDISOBind: accurate prediction of RNA-, DNA- and protein-binding intrinsically disordered residues with deep multi-task learning. Briefings in Bioinformatics 23, doi:10.1093/bib/bbab521 (2021).
27 Park, J. & Sandberg, I. W. Universal Approximation Using Radial-Basis-Function Networks. Neural Computation 3, 246-257 (1991).
28 Chen, S.-L., Cowan, C. & Grant, P. M. Orthogonal least squares learning algorithm for radial basis function networks. IEEE transactions on neural networks 2 2, 302-309 (1991).
29 Mathews, L. & Hari, S. Learning From Imbalanced Data. Advances in Computer and Electrical Engineering (2019).
30 Wahba, G. Optimal convergence properties of variable knot, kernel, and orthogonal series methods for density estimation. The Annals of Statistics, 15-29 (1975).

31 Sheather, S. J. & Jones, M. C. A reliable data-based bandwidth selection method for kernel density estimation. Journal of the royal statistical society series b-methodological 53, 683-690 (1991).
32 Zandieh, A., Han, I., Daliri, M. & Karbasi, A. KDEformer: Accelerating Transformers via Kernel Density Estimation. ArXiv abs/2302.02451 (2023).
33 Maaten, L. v. d. & Hinton, G. E. Visualizing Data using t-SNE. Journal of Machine Learning Research 9, 2579-2605 (2008).
34 Kyte, J. & Doolittle, R. F. A simple method for displaying the hydropathic character of a protein. Journal of molecular biology 157 1, 105-132 (1982).
35 Li, N., Sun, Z. & Jiang, F. Prediction of protein-protein binding site by using core interface residue and support vector machine. BMC Bioinformatics 9, 553 - 553 (2008).
36 Walls, A. C. et al. Structure, Function, and Antigenicity of the SARS-CoV-2 Spike Glycoprotein. Cell 181, 281-292.e286, doi:https://doi.org/10.1016/j.cell.2020.02.058 (2020).
37 Wrapp, D. et al. Cryo-EM structure of the 2019-nCoV spike in the prefusion conformation. Science 367, 1260-1263, doi:doi:10.1126/science.abb2507 (2020).
38 Ye, G., Liu, B. & Li, F. Cryo-EM structure of a SARS-CoV-2 omicron spike protein ectodomain. Nature Communications 13, 1214, doi:10.1038/s41467-022-28882-9 (2022).
39 He, P. et al. SARS-CoV-2 Delta and Omicron variants evade population antibody response by mutations in a single spike epitope. Nature Microbiology 7, 1635-1649, doi:10.1038/s41564-022-01235-4 (2022).
40 Lan, J. et al. Structure of the SARS-CoV-2 spike receptor-binding domain bound to the ACE2 receptor. Nature 581, 215-220, doi:10.1038/s41586-020-2180-5 (2020).
41 Koley, T., Kumar, M., Goswami, A., Ethayathulla, A. S. & Hariprasad, G. Structural modeling of Omicron spike protein and its complex with human ACE-2 receptor: Molecular basis for high transmissibility of the virus. Biochemical and Biophysical Research Communications 592, 51 - 53 (2022).
42 Shen, Z., Sang, Z. & Shi, Y. Nanobodies as a powerful platform for biomedicine. Trends in molecular medicine 28, 1006-1007, doi:10.1016/j.molmed.2022.08.007 (2022).
43 Yang, E. Y. & Shah, K. Nanobodies: Next Generation of Cancer Diagnostics and Therapeutics. Frontiers in oncology 10, 1182, doi:10.3389/fonc.2020.01182 (2020).
44 Ye, G. et al. Discovery of Nanosota-2, -3, and -4 as super potent and broad-spectrum therapeutic nanobody candidates against COVID-19. Journal of virology 97, e0144823, doi:10.1128/jvi.01448-23 (2023).
45 Berman, H. M. et al. The Protein Data Bank. Nucleic Acids Research 28, 235-242, doi:10.1093/nar/28.1.235 (2000).
46 Cerutti, G. et al. Potent SARS-CoV-2 neutralizing antibodies directed against spike N-terminal domain target a single supersite. Cell Host & Microbe 29, 819-833.e817, doi:https://doi.org/10.1016/j.chom.2021.03.005 (2021).

47 Moriyama, S. et al. Structural delineation and computational design of SARS-CoV-2-neutralizing antibodies against Omicron subvariants. Nature Communications 14, 4198, doi:10.1038/s41467-023-39890-8 (2023).
48 Gan, H. H., Zinno, J., Piano, F. & Gunsalus, K. C. Omicron Spike protein has a positive electrostatic surface that promotes ACE2 recognition and antibody escape. bioRxiv (2022).
49 Shrock, E. et al. Viral epitope profiling of COVID-19 patients reveals cross-reactivity and correlates of severity. Science 370, doi:10.1126/science.abd4250 (2020).
50 Zhang, H., Penninger, J. M., Li, Y., Zhong, N. & Slutsky, A. S. Angiotensin-converting enzyme 2 (ACE2) as a SARS-CoV-2 receptor: molecular mechanisms and potential therapeutic target. Intensive care medicine 46, 586-590, doi:10.1007/s00134-020-05985-9 (2020).
51 Goddard, T. D. et al. UCSF ChimeraX: Meeting modern challenges in visualization and analysis. Protein Science 27 (2018).
52 Vreven, T. et al. Updates to the Integrated Protein-Protein Interaction Benchmarks: Docking Benchmark Version 5 and Affinity Benchmark Version 2. J Mol Biol 427, 3031-3041, doi:10.1016/j.jmb.2015.07.016 (2015).
53 Kundrotas, P. J. et al. Dockground: A comprehensive data resource for modeling of protein complexes. Protein Sci 27, 172-181, doi:10.1002/pro.3295 (2018).
54 Zhang, C., Zhang, X., Freddolino, P. L. & Zhang, Y. BioLiP2: an updated structure database for biologically relevant ligand-protein interactions. Nucleic Acids Res 52, D404-D412, doi:10.1093/nar/gkad630 (2024).
55 Kabsch, W. & Sander, C. Dictionary of protein secondary structure: pattern recognition of hydrogen-bonded and geometrical features. Biopolymers 22, 2577-2637, doi:10.1002/bip.360221211 (1983).
56 Altschul, S. F. et al. Gapped BLAST and PSI-BLAST: a new generation of protein database search programs. Nucleic Acids Res 25, 3389-3402, doi:10.1093/nar/25.17.3389 (1997).
57 Suzek, B. E., Huang, H., McGarvey, P., Mazumder, R. & Wu, C. H. UniRef: comprehensive and non-redundant UniProt reference clusters. Bioinformatics 23, 1282-1288, doi:10.1093/bioinformatics/btm098 (2007).
58 Remmert, M., Biegert, A., Hauser, A. & Söding, J. HHblits: lightning-fast iterative protein sequence searching by HMM-HMM alignment. Nat Methods 9, 173-175, doi:10.1038/nmeth.1818 (2011).
59 Mirdita, M. et al. Uniclust databases of clustered and deeply annotated protein sequences and alignments. Nucleic Acids Research 45, D170-D176, doi:10.1093/nar/gkw1081 (2016).
60 Ioffe, S. & Szegedy, C. Batch Normalization: Accelerating Deep Network Training by Reducing Internal Covariate Shift. ArXiv abs/1502.03167 (2015).
61 Nair, V. & Hinton, G. E. in International Conference on Machine Learning.
62 Srivastava, N., Hinton, G. E., Krizhevsky, A., Sutskever, I. & Salakhutdinov, R. Dropout: a simple way to prevent neural networks from overfitting. J. Mach. Learn. Res. 15, 1929-1958 (2014).
63 Vaswani, A. et al. in Neural Information Processing Systems.

64 Qi, C., Yi, L., Su, H. & Guibas, L. J. in Neural Information Processing Systems.
65 Ingraham, J., Garg, V. K., Barzilay, R. & Jaakkola, T. in DGS@ICLR.
66 Igashov, I., Pavlichenko, N. & Grudinin, S. Spherical convolutions on molecular graphs for protein model quality assessment. Mach. Learn. Sci. Technol. 2, 45005 (2020).
67 Lin, T.-Y., Goyal, P., Girshick, R. B., He, K. & Dollár, P. Focal Loss for Dense Object Detection. 2017 IEEE International Conference on Computer Vision (ICCV), 2999-3007 (2017).

Click here to access/download
Supplementary Material
Supplementary Material of GraphRBF.pdf

Cover Letter
Click here to access/download;Personal Cover;Cover_letter.docx

Dear Editor,

We are submitting the enclosed manuscript, entitled "**Protein-protein and protein-nucleic acid binding site prediction via interpretable hierarchical geometric deep learning**" to ***GigaScience*** for your consideration. This paper has passed the presub discussion.

The accurate identification of protein-protein and protein-nucleic acid binding sites is pivotal for understanding protein functions and aiding disease diagnosis and drug design. However, computational prediction of binding sites remains a highly challenging task due to the limited knowledge of residue binding patterns. Here, we introduce GraphRBF, an innovative hierarchical geometric deep learning model that addresses the challenge by comprehensively characterizing residue binding patterns learned from big data. The key contributions of our work are summarized as follows.

(1) GraphRBF characterizes the binding pattern of a residue by the spatial distribution of its neighboring residues combined with the physicochemical information interactions among the residues in the local environment.

(2) GraphRBF describes physicochemical information interactions by designing an enhanced graph neural network and represents local residue spatial distributions by introducing a prioritized radial basis function neural network.

After training and testing, GraphRBF outperforms state-of-the-art methods in predicting protein-protein, protein-DNA, and protein-RNA binding sites across various benchmark datasets. In addition, we apply GraphRBF on the glycoprotein of SARS-CoV-2 omicron variant for the predictions of epitopes. As a result, it not only successfully predicts known binding regions and important binding sites with different antibodies on the NTD, RBD, SD1, and SD2 regions, but also identifies multiple potential antibody binding regions and receptor binding sites supported by strong biological evidence. To facilitate the broader application of GraphRBF, we have developed an user friendly web server available at http://liulab.top/GraphRBF/server, providing researchers with a robust tool for identifying binding sites in their proteins of interest.

Thank you for your time and consideration. If you have any questions, please do not hesitate to contact me.

Yours sincerely,

Juntao Liu, Ph.D.
School of Statistics and Mathematics, Shandong University
E-mail: juntaosdu@126.com
