# Supplementary material for: Protein–protein and protein–nucleic acid binding site prediction via interpretable hierarchical geometric deep learning
Source: Gigascience. 2024 Nov 1;13:giae080. doi: 10.1093/gigascience/giae080 (PMC11528319; doi:10.1093/gigascience/giae080)
Supplement: giae080_Supplemental_File [file giae080_supplemental_file.pdf]

Supplementary Material

Protein-protein and protein-nucleic acid binding site prediction via interpretable hierarchical geometric deep learning

Shizhuo Zhang[1,†], Jiyun Han[1,†], Juntao Liu[1,*]

[1]School of Mathematics and Statistics, Shandong University (Weihai), Weihai, 264209, China

[†]These authors contributed equally to this study

[*]To whom correspondence should be addressed

Email addresses:

JL: juntaosdu@126.com

Table of Content

Supplementary Methods

Supplementary Tables

Supplementary Figures

Supplementary Methods

Method S1: Evaluation metrics

In this section, we use multiple classification evaluation metrics commonly used in binary classification tasks to compare the performance of GraphRBF with other methods. The evaluation metrics include Accuracy (ACC), Recall (Rec), Precision (Pre), F1-Score(F1), Matthews Correlation Coefficient (MCC), Area Under the Receiver Operating Characteristic Curve (AUROC), and Area Under the Precision-Recall Curve (AUPRC).

The Rec, Pre, F1 and MCC are calculated as follows:

$$\mathrm{ACC} = \frac{\mathrm{TP+TN}}{\mathrm{TP + FP+TN+FN}}$$

$$\mathrm{Pre} = \frac{\mathrm{TP}}{\mathrm{TP + FP}}$$

$$\mathrm{Rec} = \frac{\mathrm{TP}}{\mathrm{TP + FN}}$$

$$\mathrm{F1} = 2 \times \frac{\mathrm{Pre}\times\mathrm{Rec}}{\mathrm{Pre + Rec}}$$

$$\mathrm{MCC} = \frac{\mathrm{TP}\times\mathrm{TN} - \mathrm{FP}\times\mathrm{FN}}{\sqrt{(\mathrm{TP + FP})(\mathrm{TP + FN})(\mathrm{TN + FP})(\mathrm{TN + FN})}}$$

where TP represents true positives, TN represents true negatives, FP represents false positives, and FN represents false negatives.

The AUROC and AUPRC are computed based on the Receiver Operating Characteristic (ROC) curve and Precision-Recall curve, respectively. The ROC curve plots the True Positive Rate (TPR) against the False Positive Rate (FPR) at various threshold settings, while the Precision-Recall curve plots Precision against Recall. The AUROC represents the area under the ROC curve, and the AUPRC represents the area under the Precision-Recall curve.We compute these evaluation metrics on our test dataset to assess the effectiveness of our model in classifying instances of interest.

Method S2: Details of component ablation experiments

We conduct ablation experiments on different components of the model. We retrain the model and compare the performance by removing the EGNN, RBFNN and attention of kernels in RBFNN, respectively on the protein-binding protein test set. Here are some details on how to do it:

When using EGNN alone, we generate graph features by the aggregation of node features as follows.

$$u^0 = MLP_u^0\left(\left[\sum_{i=1}^{N_{residues}} v_i^0\right]\right)$$

$$\tilde{u}^k = MLP_u^k\left(\left[u^{k-1} \; ; \; \sum_{i=1}^{N_{residues}} v_i^k\right]\right)$$

$$u^k = u^{k-1} + \tilde{u}^k$$

When using RBFNN alone, we encode the node features by using only the node feature vectors and local position information vectors as follows.

$$v_i^0 = MLP_v^0([v_i \, ; \, p_i])$$

$$\tilde{v}_i^k = MLP_v^k\left(\left[v_i^{k-1} \, ; \, p_i^{k-1} \; ; \, u^{k-1}\right]\right)$$

$$v_i^k = v_i^{k-1} + \tilde{v}_i^k$$

When using the RBFNN without attention in combination with the EGNN, we aggregate the node features by a direct summation rather than a weighted summation using the attention coefficients as weights.

$$MLP_m^0(X) = BN\left(W_2^0\left(\text{Dropout}\left(\text{ReLU}\left(\text{BN}\left(W_1^0 X + b_1^0\right)\right)\right)\right) + b_2^0\right)$$

$$y_m^0 = MLP_m^0\left\{\sum_{g=1}^{G}\left[\sum_{i=1}^{N_{residues}} \mathcal{g}_g^0\left(\mu_g^0, \sigma_g^0, p_i\right)\check{v}_i^0\right]\right\}$$

$$MLP_m^k(X) = BN\left(W_2^k\left(\text{Dropout}\left(\text{ReLU}\left(\text{BN}\left(W_1^k X + b_1^k\right)\right)\right)\right) + b_2^k\right)$$

$$y_m^k = MLP_m^k\left\{\sum_{g=1}^{G}\left[\sum_{i=1}^{N_{residues}} \mathcal{g}_{gi}^k \check{v}_i^k\right]\right\}$$

For details on the various parameters described above, please refer to the **Methods** section.

Method S3: Definition of radial basis function in ablation experiments

Here, we show the four radial basis functions in the RBFNN module tested in Radial basis function ablation experiments.

Gentle Decay Gaussian-like kernel function (GDG) $g(\mu, \sigma, p) = \exp\left(-\ \|p - \mu\|/2\sigma^2\right)$

The original Gaussian function $\mathcal{G}(\mu, \sigma, p) = \exp\left(-\ \|p - \mu\|^2/2\sigma^2\right)$

Inverse quadratic function $\varphi(\mu, \sigma, p) = \left(1 + (p - \mu)^2/\sigma^2\right)^{-1}$

Inverse multiquadratic function $\phi(\mu, \sigma, p) = \left(1 + (p - \mu)^2/\sigma^2\right)^{-\frac{1}{2}}$

Method S4: Details of dataset curation

In this section, we introduce the datasets used in our work. In order to fairly assess the performance of our model and compare it with other methods, the protein-protein interaction datasets were obtained from Protein-Protein Docking Benchmark 5.5[1] (DBD 5.5) and Dockground[2]. These datasets consist of non-redundant, high-resolution structures, including both bound and unbound structures for each protein complex. Considering that protein conformation tends to change to varying degrees when interacting with other molecules in the cell, it makes more sense to use the unbound structures prior to the interaction to train and test the model. Finally, we obtained 1,251 protein-binding proteins as a dataset, and then divided the data into a training dataset and a test dataset at a ratio of 0.2. In addition, we further divided the training data into a training dataset and a validation dataset, which were specifically used for hyper-parameter tuning.

We constructed a baseline dataset of nucleic acid binding proteins from the BioLiP2[3] database, which collects biologically relevant ligand-protein interactions that are structurally resolved in complexes. DNA/RNA-binding proteins were collected from the dataset as of October 7, 2023, and contain a total of 42,457 DNA-protein complex entries and 146,306 RNA-protein complex entries. First, we selected high-resolution structures with a resolution of less than 8.0 Å. The resolution can reflect the accuracy of protein structure analysis; higher resolution indicates finer protein structure, while lower resolution indicates that the atomic structure in the crystal is more difficult to be clearly resolved and localized. Next, sequence similarity clustering was performed on the redundant data using psi-cd-hit[4], and the structures with the highest resolution were selected as representative entries for each cluster. Finally, we kept 1,871 RNA-binding proteins and 1,101 DNA-binding proteins, after which we randomly divided the test set using a ratio of 0.2, and then divided the training and validation sets.

Eventually we obtained 1001 protein-binding proteins, 881 DNA-binding proteins and 1497 RNA-binding proteins as the training set; 250 protein-binding proteins, 220 DNA-binding proteins and 374 RNA-binding proteins as the test set. The details of the dataset are summarized in Table 2.

Method S5: Details of node features extraction

We extract multiple types of sequence-based and structure-based amino acid features as inputs to the model, including atomic features of residues, secondary structure encoding, evolutionary information, and residue type encoding.

Atomic features of the residues. We extract seven atomic features for each heavy atom belonging to the target residue based on the PDB file: atom mass, B-factor, whether it is a residue side-chain atom, electronic charge, the number of hydrogen atoms bonded to it, whether it is in a ring, and the van der Waals radius of the atom. Since different residues may have different numbers of atoms, we average the atomic features of all atoms in each dimension as the seven-dimensional atomic features of the target amino acid:

$$x_i = \frac{1}{N_a}\left(\sum_{j=1}^{N_a} f_{i,j}\right)$$

where N_a is the number of atoms contained in the target amino acid, and x_i, $f_{i,j}$ are the i-th(i=1,2,…,7) atomic feature of the amino acid and atom, respectively. Finally we extracted the atomic feature matrix of size L×7 for the protein with L residues.

The secondary structure profile of the residue. We use DSSP[5] to generate secondary structure features of residues including one column of residue water-exposed surface, five columns of bond and torsion angles and eight columns of one-hot encoded secondary structure with eight states. The eight states of secondary structure contain B(residue in isolated β-bridge), E(extended strand, participates in β-ladder), G(310-helix), H(α-helix), I(π-helix), S(bend), T(H-bonded turn) and others. Finally, the secondary structure encoding matrix of size L×14 is obtained.

Evolutionary information. PSI-BLAST profile and the HHblits profile have different back-end algorithms for searching databases, so the features extracted by them are important and complementary. PSI-BLAST[6] applies the heuristic algorithms and dynamic programming to search the Uniref90 database[7] for homologous sequences with three iterations and *e-value*<10^{-3}. The generated position-specific scoring matrix (PSSM) has a size of L×20, after which we also normalize its element x by a sigmoid function:

$$\bar{x} = \frac{1}{1+e^{-x}}$$

HHblits[8] is based on a Hidden Markov Model (HMM) for searching the uniclust30 database[9] using default parameters to generate an HMM matrix of the query sequence. The HMM matrix has a size of L×30, including 20 columns of observed frequencies for 20 amino acids in homologous sequences, seven columns of transition frequencies and three columns of local diversities. Each element x is normalized by:

$$\bar{x} = \frac{x}{10000}$$

Amino acid type encoding. First, the 20 types of amino acids are ordered as ACDEFGHIKLMNPQRSTVWY, and then a 20-dimensional vector [v_1, v_2,..., v_{20}] is constructed for each residue a in protein, where $v_i = 1$ if a belongs to the i-th type of amino acid and $v_i = 0$ otherwise. In this way, we obtain one-hot coding of amino acid types of size L×20.

In conclusion, we extract a feature matrix of size L×91 for each protein, followed by min-max normalization for each column of the feature matrix:

$$\bar{x} = \frac{x - x_{min}}{x_{max} - x_{min}}$$

x_{min} and x_{max} are the minimum and the maximum values of this feature in the training set, respectively.

Method S6: Advantage analysis of FGM in PRBFNN

The feature extraction using multiple filters mentioned above has several advantages over the general multilayer perceptron[10-12] or spherical harmonic function[13]. First, we use GDG weights g_g^0 calculated based on local coordinates to aggregate features across nodes rather than using the same weights to treat each node equally or using learnable parameters, and we argue that this aggregation pattern is directly related to the spatial distribution of the nodes in a local neighborhood. Second, our filter is a kind of skillful combination of RBF and MLP, and the combination may give better results than using them alone[11,14,15]. The idea is to first aggregate the node features based on the GDG kernel weights to get a representation of each GDG kernel, then aggregate the features of GDG kernels and use the dimensionality transformation, activation operation, and normalization in MLP to get the output of one filter. The introduction of the attentional mechanism also allows the model to notice important GDG kernels. Our final feature extraction result is a concatenation of multiple filter outputs, which enhances the representational power of our model. Third, because the GDG functions decay rapidly as $||p - \mu|| \to \infty$, our filter has an almost compact support, which ensures that the features of the GDG kernel reflect the distance from the nodes to the center of the kernel. The center μ and the width σ of each GDG kernel are set as learnable parameters, which makes the model learning process more flexible. Fourth, we use an outer product $\otimes$ for feature extraction, which converts the representation from the feature vectors of a variable number $N_{residues}$ of nodes to the feature vectors of a fixed number G of GDG kernels. By this approach we obtain a higher level feature representation that incorporates information about the spatial distribution of nodes and the node feature vectors, and then we can use some models that restrict the fixed size, such as CNN[16,17], RNN[18,19], attention mechanisms[20,21] and so on.

Method S7: Introduction of Focal Loss

Considering that there is a serious imbalance of positive and negative samples in the binding site data, we use Focal Loss[22], which addresses this class imbalance by reshaping the standard cross entropy loss such that it down-weights the loss assigned to well-classified examples. The novel Focal Loss focuses training on a sparse set of hard examples and prevents the vast number of easy negatives from overwhelming the detector during training(see details in Supplemental Methods S7). The focal loss is formulated as follows:

$$\mathrm{FL}(p_t) =- \alpha_t(1-p_t)^{\gamma}\log(p_t)$$

where p_t is the classification probability of a particular class, $(1-p_t)^{\gamma}$ is a modulating factor to the cross entropy loss, with tunable focusing parameter $\gamma \geq 0$ to adjust the weight of hard and easy samples, and $\alpha_t \in [0,1]$ is also a parameter, which denotes classification weighting factor, to give the weight of minority and majority samples. For the parameters here we choose the best-case settings given in the original paper: $\alpha_t = 0.25$ and $\gamma = 2$. Our study leveraged the Focal Loss function to address the challenge posed by the significant imbalance between positive and negative samples in our binding site datasets. Focal Loss effectively focuses the model's learning effort on the harder-to-classify positive samples, thereby enhancing the detection accuracy of less prevalent, yet critical, binding sites. This approach not only mitigated the impact of class imbalance on our model's performance but also ensured a more robust and discriminative learning process.

Supplementary Tables

Table S1. Ablation studies on protein-DNA binding test set DNA-220_Test with different settings of hyperparameters

	r[a]	d[b]	G[c]	N_f[d]	K[e]	D_p[f]	D[g]	Rec	Pre	F1	MCC	AUC	PRC
Base	20	10	32	64	1	64	128	**0.581**	0.320	**0.412**	**0.403**	**0.920**	**0.357**
A	15							0.518	0.318	0.394	0.378	0.919	0.335
	25							0.559	0.317	0.405	0.393	0.921	0.345
B		5						0.492	0.347	0.407	0.388	0.921	0.349
		15						0.523	0.332	0.406	0.390	0.916	0.351
C			16					0.479	0.348	0.403	0.382	0.919	0.346
			64					0.448	0.354	0.396	0.373	0.914	0.338
D				32				0.471	0.336	0.392	0.37	0.919	0.336
				128				0.437	0.347	0.387	0.363	0.917	0.314
E					0			0.477	0.350	0.404	0.383	0.918	0.348
					2			0.488	0.333	0.396	0.377	0.919	0.331
					4			0.457	0.338	0.388	0.367	0.917	0.314
F						3		0.548	0.324	0.407	0.394	0.918	0.351
						32		0.514	0.332	0.403	0.386	0.912	0.333
						128		0.477	0.340	0.397	0.376	0.915	0.335
G							64	0.446	**0.375**	0.407	0.384	0.920	0.348
							256	0.447	0.360	0.399	0.376	0.917	0.335
							512	0.503	0.327	0.396	0.378	0.919	0.337

[a]Radius of the structural neighborhood: it defines the point cloud within the neighborhood of the target residue, and its unit is Å.

[b]The distance threshold to get the adjacency matrix: it defines the threshold for concatenating edges between residues, and its unit is Å.

[c]The number of Gaussian kernels in each filter.

[d]The number of filters used in PRBFNN.

[e]The number of representation layer. Layer 0 stands for the encoder layer.

[f]The dimension D_p of encoded position information vector using local coordinates.

[g]The dimension of encoded node, edge and graph feature vector. We set $D_\mathrm{e} = D_\mathrm{v} = D_\mathrm{u}$.

Table S2. Ablation studies on protein-RNA binding test set RNA-374_Test with different settings of hyperparameters

	r[a]	d[b]	G[c]	N_f[d]	K[e]	D_p[f]	D[g]	Rec	Pre	F1	MCC	AUC	PRC
Base	20	10	32	64	1	64	128	**0.596**	**0.465**	**0.522**	**0.481**	**0.902**	**0.512**
A	15							0.550	0.474	0.509	0.465	0.895	0.500
	25							0.573	0.468	0.515	0.473	0.898	0.507
B		5						0.573	0.472	0.518	0.475	0.900	0.51
		15						0.606	0.451	0.517	0.476	0.903	0.508
C			16					0.579	0.464	0.515	0.473	0.900	0.508
			64					0.583	0.449	0.507	0.464	0.895	0.495
D				32				0.571	0.451	0.504	0.461	0.891	0.493
				128				0.568	0.442	0.497	0.453	0.890	0.485
E					0			0.562	0.477	0.516	0.473	0.901	0.510
					2			0.598	0.443	0.509	0.467	0.896	0.500
					4			0.551	0.488	0.494	0.450	0.889	0.482
F						3		0.569	0.477	0.519	0.476	0.904	0.509
						32		0.604	0.451	0.516	0.475	0.898	0.506
						128		0.587	0.435	0.500	0.457	0.892	0.491
G							64	0.575	0.474	0.519	0.477	0.901	0.507
							256	0.566	0.469	0.513	0.470	0.901	0.509
							512	0.606	0.438	0.508	0.467	0.894	0.494

[a]Radius of the structural neighborhood: it defines the point cloud within the neighborhood of the target residue, and its unit is Å.

[b]The distance threshold to get the adjacency matrix: it defines the threshold for concatenating edges between residues, and its unit is Å.

[c]The number of Gaussian kernels in each filter.

[d]The number of filters used in PRBFNN.

[e]The number of representation layer. Layer 0 stands for the encoder layer.

[f]The dimension D_p of encoded position information vector using local coordinates.

[g]The dimension of encoded node, edge and graph feature vector. We set $D_\mathrm{e} = D_\mathrm{v} = D_\mathrm{u}$.

Table S3. The specifics of each of the component ablation experiments on the three test sets

Datasets	Settings	Rec	Pre	F1	MCC	AUC	PRC	time
P-250_Test	EGNN	0.396	0.278	0.327	0.262	0.805	0.264	24%
	RBFNN	0.5	0.215	0.301	0.238	0.778	0.229	53%
	EGNN+RBFNN(noattention)	0.422	0.324	0.366	0.306	0.821	0.306	76%
	EGNN+RBFNN	0.43	0.367	0.396	0.341	0.825	0.366	100%
	Inverse Quadratic	0.405	0.377	0.391	0.336	0.82	0.357	97%
	Inverse Multiquadratic	0.406	0.371	0.387	0.332	0.824	0.353	98%
	Gaussian	0.432	0.346	0.385	0.327	0.824	0.35	101%
	GDG	0.43	0.367	0.396	0.341	0.825	0.366	100%
DNA-220_Test	EGNN	0.395	0.305	0.344	0.32	0.89	0.265	27%
	RBFNN	0.423	0.265	0.326	0.303	0.879	0.244	51%
	EGNN+RBFNN(noattention)	0.557	0.299	0.389	0.379	0.913	0.322	78%
	EGNN+RBFNN	**0.581**	**0.32**	**0.412**	**0.403**	**0.92**	**0.357**	100%
	Inverse Quadratic	0.546	0.318	0.402	0.389	0.916	0.351	96%
	Inverse Multiquadratic	0.527	0.318	0.397	0.381	0.916	0.347	98%
	Gaussian	0.514	0.344	0.405	0.387	0.921	0.349	102%
	GDG	**0.581**	**0.32**	**0.412**	**0.403**	**0.92**	**0.357**	100%
RNA-374_Test	EGNN	0.507	0.398	0.446	0.396	0.853	0.411	23%
	RBFNN	0.51	0.382	0.439	0.386	0.845	0.386	54%
	EGNN+RBFNN(noattention)	0.557	0.43	0.486	0.44	0.882	0.458	75%
	EGNN+RBFNN	**0.596**	**0.465**	**0.522**	**0.481**	**0.902**	**0.512**	100%
	Inverse Quadratic	0.54	0.49	0.514	0.471	0.899	0.509	99%
	Inverse Multiquadratic	0.579	0.453	0.508	0.465	0.896	0.504	99%
	Gaussian	0.3571	0.466	0.513	0.471	0.901	0.506	101%
	GDG	**0.596**	**0.465**	**0.522**	**0.481**	**0.902**	**0.512**	100%

Table S4. Summary of the benchmark datasets

Type	Dataset	$N_{protein}$[a]	N_{pos}[b]	N_{neg}[c]	PNratio[d]
Protein	P-1001_Train	1001	25172	264564	0.095
	P-250_Test	250	6386	73391	0.087
DNA	DNA-881_Train	881	9962	251759	0.04
	DNA-220_Test	220	2537	68401	0.037
RNA	RNA-1497_Train	1497	33444	384358	0.087
	RNA-374_Test	374	9331	110259	0.085

[a]Number of proteins.

[b]Number of binding residues.

[c]Number of non-binding residues.

[d]PNratio= N_{pos}/N_{neg}.

Supplementary Figures

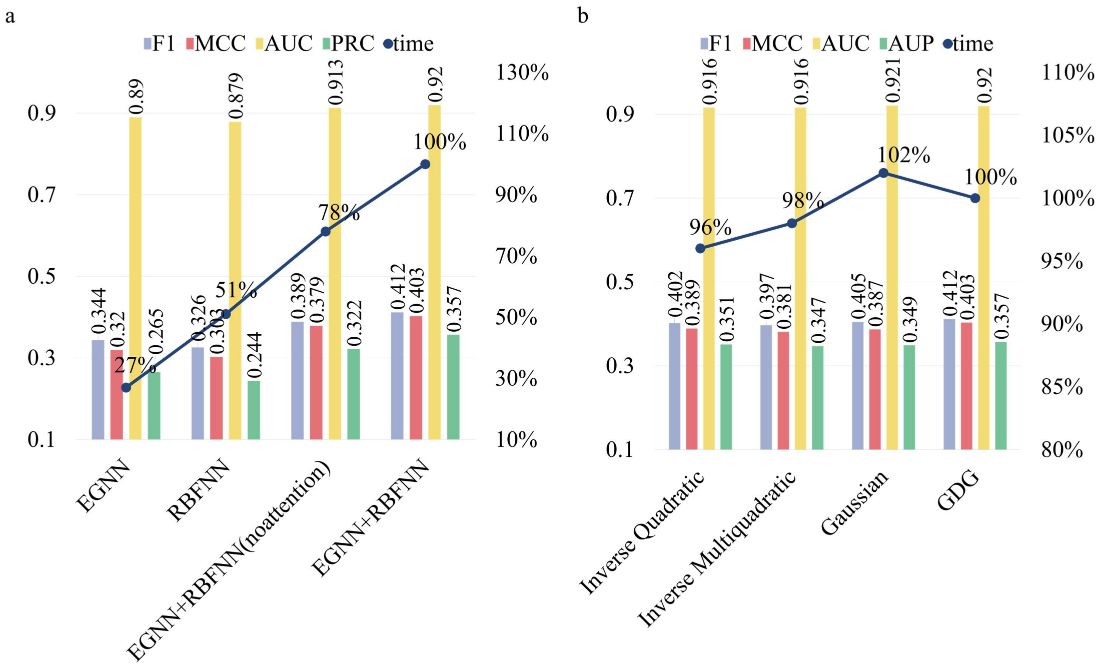

Fig. S1 Component and feature ablation experiments on DNA-220_Test. **a,** Performance of GraphRBF with different combinations of components. **b,** Performance of GraphRBF with different kernel function using in filter.

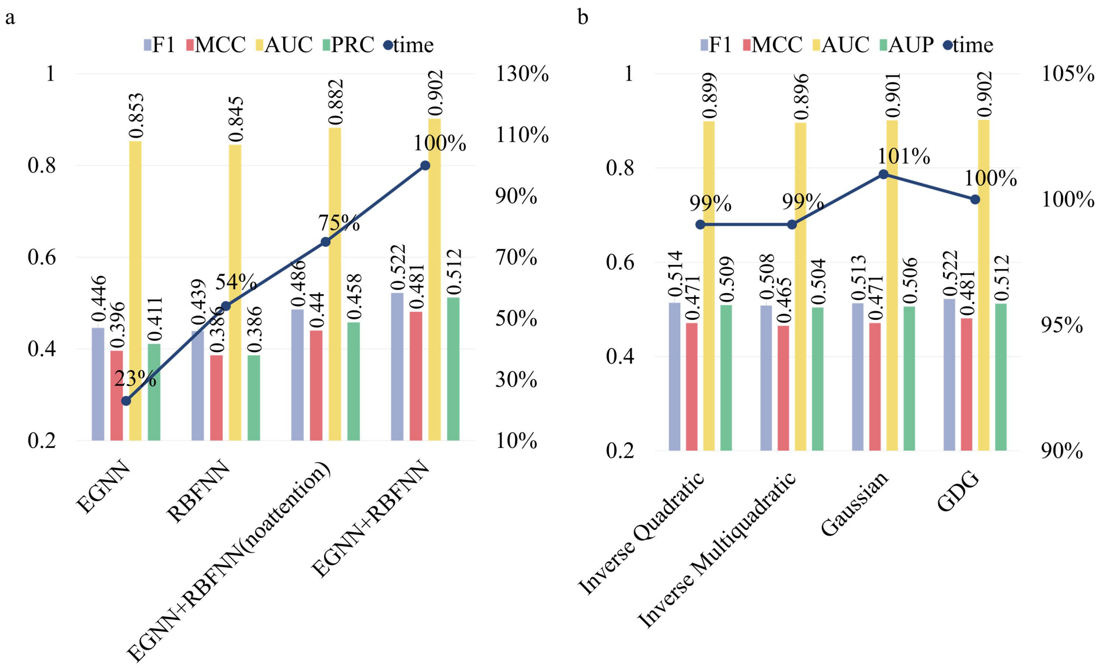

Fig. S2 Component and feature ablation experiments on RNA-374_Test. **a,** Performance of GraphRBF with different combinations of components. **b,** Performance of GraphRBF with different kernel function using in filter.

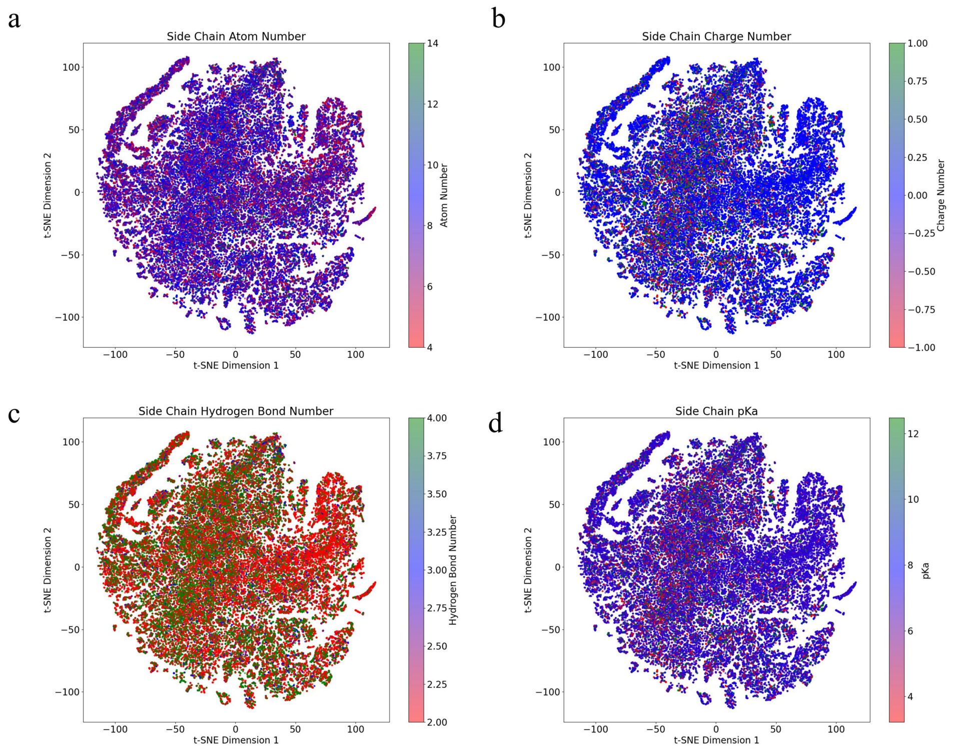

Fig. S3 Visualization of representations on physicochemical properties. The two-dimensional projection visualisation of the extracted graph feature using t-SNE. Colouring t-SNE projection based on atomic number(**a**), charge(**b**), number of hydrogen bonds(**c**), and side chain pKa(**d**).

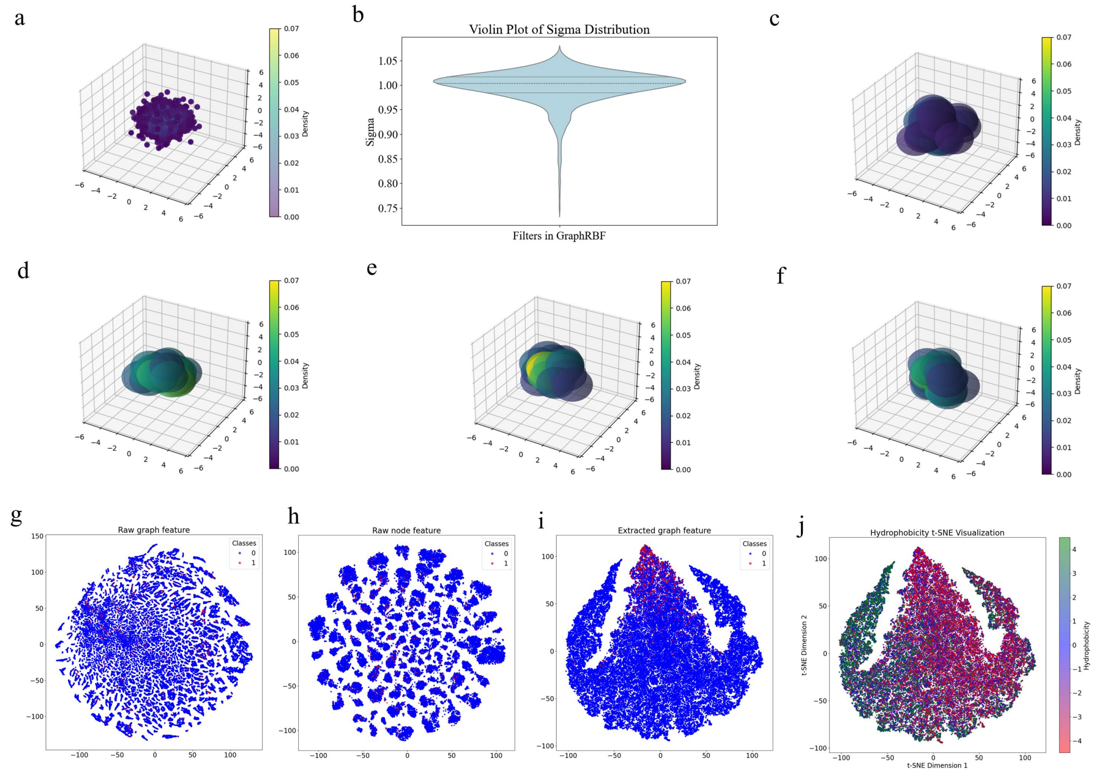

Fig. S4 Interpretation and visualization results of DNA-220_Test.

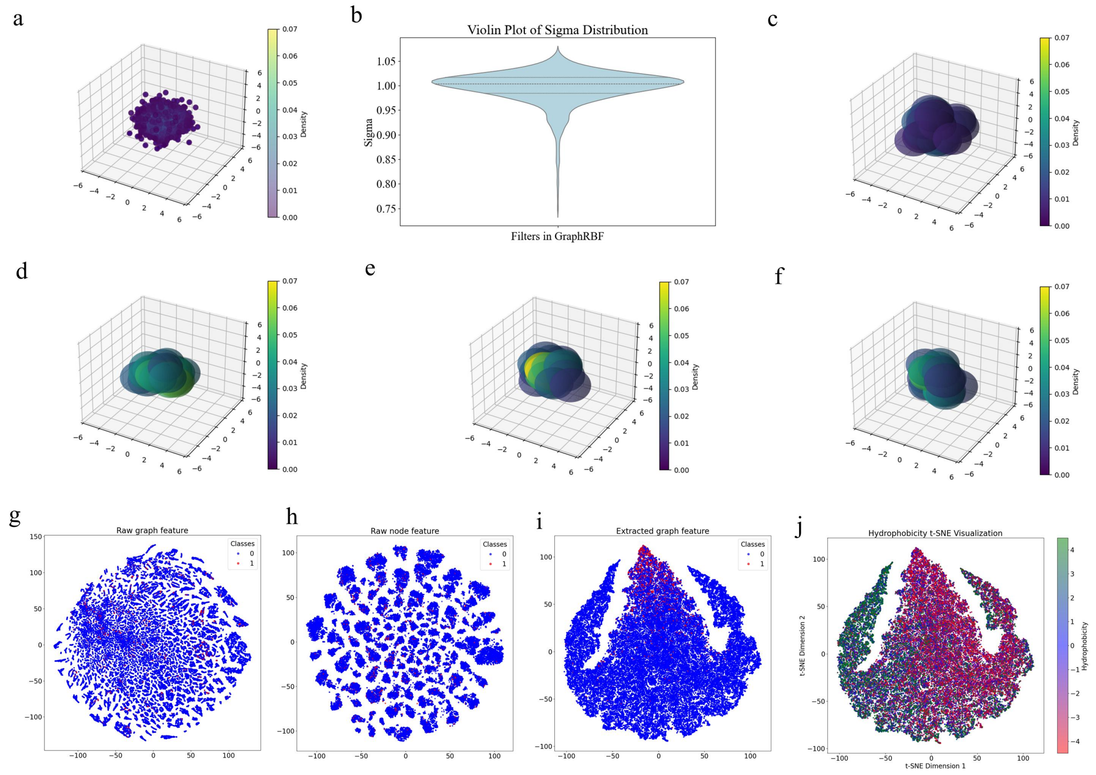

Fig. S5 Interpretation and visualization results of RNA-374_Test.

References

1 Vreven, T. et al. Updates to the Integrated Protein-Protein Interaction Benchmarks: Docking Benchmark Version 5 and Affinity Benchmark Version 2. J Mol Biol 427, 3031-3041, doi:10.1016/j.jmb.2015.07.016 (2015).

2 Kundrotas, P. J. et al. Dockground: A comprehensive data resource for modeling of protein complexes. Protein Sci 27, 172-181, doi:10.1002/pro.3295 (2018).

3 Zhang, C., Zhang, X., Freddolino, P. L. & Zhang, Y. BioLiP2: an updated structure database for biologically relevant ligand-protein interactions. Nucleic Acids Res 52, D404-D412, doi:10.1093/nar/gkad630 (2024).

4 Yao, L., Wang, H., Song, Y. & Sui, G. BioQueue: a novel pipeline framework to accelerate bioinformatics analysis. Bioinformatics 33, 3286-3288, doi:10.1093/bioinformatics/btx403 (2017).

5 Kabsch, W. & Sander, C. Dictionary of protein secondary structure: pattern recognition of hydrogen-bonded and geometrical features. Biopolymers 22, 2577-2637, doi:10.1002/bip.360221211 (1983).

6 Altschul, S. F. et al. Gapped BLAST and PSI-BLAST: a new generation of protein database search programs. Nucleic Acids Res 25, 3389-3402, doi:10.1093/nar/25.17.3389 (1997).

7 Suzek, B. E., Huang, H., McGarvey, P., Mazumder, R. & Wu, C. H. UniRef: comprehensive and non-redundant UniProt reference clusters. Bioinformatics 23, 1282-1288, doi:10.1093/bioinformatics/btm098 (2007).

8 Remmert, M., Biegert, A., Hauser, A. & Söding, J. HHblits: lightning-fast iterative protein sequence searching by HMM-HMM alignment. Nat Methods 9, 173-175,

doi:10.1038/nmeth.1818 (2011).
9 Mirdita, M. et al. Uniclust databases of clustered and deeply annotated protein sequences and alignments. Nucleic Acids Research 45, D170-D176, doi:10.1093/nar/gkw1081 (2016).
10 Ingraham, J., Garg, V. K., Barzilay, R. & Jaakkola, T. in DGS@ICLR.
11 Qi, C., Yi, L., Su, H. & Guibas, L. J. in Neural Information Processing Systems.
12 Qi, C., Su, H., Mo, K. & Guibas, L. J. PointNet: Deep Learning on Point Sets for 3D Classification and Segmentation. 2017 IEEE Conference on Computer Vision and Pattern Recognition (CVPR), 77-85 (2016).
13 Igashov, I., Pavlichenko, N. & Grudinin, S. Spherical convolutions on molecular graphs for protein model quality assessment. Mach. Learn. Sci. Technol. 2, 45005 (2020).
14 Jiang, Q., Zhu, L., Shu, C. & Sekar, V. An efficient multilayer RBF neural network and its application to regression problems. Neural Computing and Applications 34, 4133 - 4150 (2021).
15 Chen, W. et al. Deep RBFNet: Point Cloud Feature Learning using Radial Basis Functions. ArXiv abs/1812.04302 (2018).
16 Ren, S., He, K., Girshick, R. B. & Sun, J. Faster R-CNN: Towards Real-Time Object Detection with Region Proposal Networks. IEEE Transactions on Pattern Analysis and Machine Intelligence 39, 1137-1149 (2015).
17 Krizhevsky, A., Sutskever, I. & Hinton, G. E. ImageNet classification with deep convolutional neural networks. Communications of the ACM 60, 84 - 90 (2012).
18 Mikolov, T., Karafiát, M., Burget, L., Černocký, J. H. & Khudanpur, S. in Interspeech.
19 Sak, H., Senior, A. W. & Beaufays, F. in Interspeech.
20 Vaswani, A. et al. in Neural Information Processing Systems.
21 Woo, S., Park, J., Lee, J.-Y. & Kweon, I.-S. CBAM: Convolutional Block Attention Module. ArXiv abs/1807.06521 (2018).
22 Lin, T.-Y., Goyal, P., Girshick, R. B., He, K. & Dollár, P. Focal Loss for Dense Object Detection. 2017 IEEE International Conference on Computer Vision (ICCV), 2999-3007 (2017).
