# Supplementary material for: Protein–protein and protein–nucleic acid binding site prediction via interpretable hierarchical geometric deep learning
Source: Gigascience. 2024 Nov 1;13:giae080. doi: 10.1093/gigascience/giae080 (PMC11528319; doi:10.1093/gigascience/giae080)
Supplement: giae080_Response_to_Reviewer_Comments_Original_Submission [file giae080_response_to_reviewer_comments_original_submission.pdf]

Author's Response To Reviewer Comments

Reviewer1 :
This paper introduces a novel hierarchical geometric deep learning model, GraphRBF, designed to predict protein-protein and protein-nucleic acid binding sites. The model demonstrates significant improvements over existing state-of-the-art methods in terms of accuracy and interpretability. The detailed experiments and thorough data analysis provided in the manuscript convincingly showcase the efficacy and novelty of the proposed model. However, I have some serious concerns. Please see below for details.

Comment 1: Could you specify the tools and parameters used for extracting atomic features, secondary structure features, and evolutionary information? How are these features integrated into the model inputs? During the feature extraction process, were any feature selection or dimensionality reduction techniques employed? If so, please describe these steps and the methods used in detail. Additionally, please provide a comprehensive overview of the data format inputted into the model.
Response: Thank you for the comment. For atomic features, we used PDB files to extract seven types of features for each heavy atom: atom mass, B-factor, side-chain status, electronic charge, hydrogen bond count, ring status, and van der Waals radius. Secondary structure features were generated using DSSP, which includes residue water-exposed surface, five bond and torsion angles, and eight one-hot encoded secondary structure states. For the evolutionary information, PSI-BLAST is used to search the Uniref 90 database for homologous sequences with three iterations and e-value<0.001 to generate the position-specific scoring matrix (PSSM) with 20 dimensions. HHblits is implemented to search the uniclust30 database to generate an HMM matrix of the query sequence with 30 dimensions. For the residue type encoding, the 20 types of amino acids are ordered as ACDEFGHIKLMNPQRSTVWY, and an 20-dimension one-hot coding of each residue is generated accordingly. All these features were integrated into a node feature matrix of size L×91, where L is the protein length. Min-max normalization was applied before training, but no additional feature selection or dimensionality reduction was performed. And the details of features were shown in Supplemental Method S5. In our model, each residue is treated as a sample instead of a protein. For each residue, the final input is its neighborhoods with their own node features and local coordinates, forming a comprehensive representation for the model.

Comment 2: What criteria were used to select the radius and the number of neighbors when constructing the local graph? How do these parameters affect the model's performance?
Response: Thank you for the comment. We selected a radius of 20 Å and an adjacency matrix threshold of 10 Å to define the local neighborhood and edge connections, respectively. These parameters were determined through preliminary experiments to optimize the balance between capturing local context and computational efficiency. Experiments A and B in Table 2 demonstrate that a smaller radius, while reducing computational load, can compromise the model's performance by limiting the sensory field. Conversely, larger values enhance context capture but increase computational demand. Our choices consider both the typical ranges of intermolecular interactions, such as covalent bonds, hydrogen bonds, and van der Waals forces, and the need to maintain a manageable computational workload. We believe this balance is crucial for the model's effectiveness.

Comment 3: Could you elaborate on the details of constructing the local coordinate system? For instance, when selecting the first direction vector and the second direction vector, were other possible choices considered?
Response: Thank you for the comment. Our approach is based on the α-carbon coordinates from the PDB file, serving as pseudo-positions for amino acids. The local coordinate system is established with the target residue as the origin. The first direction vector f1 is determined by its closest neighbor, and the second direction vector f2 by its side chain centroid, ensuring invariance to rotation and translation. And then, The third and fourth direction vectors f3 and f4 are then constructed by cross-producting f1 with f2, and f1 with f3 as follows.
$f3=(f1\times f2)/||f1\times f2||$
$f3=(f1\times f3)/||f1\times f3||$
Finally, the x, y, and z axes of the local coordinate system are respectively defined as the direction vectors f4, f2, and f3. We explored alternative choices for the first and second direction vectors, including using the

main chain center and the entire amino acid center. After evaluating various permutations and combinations, we selected the configuration that most effectively captured local information and delivered the optimal results.

Comment 4: The manuscript mentions numerous advantages of the radial basis function model. Was there a specific analysis or explanation provided for these advantages? Additionally, what considerations led to the choice of using a radial basis function neural network to extract spatial distribution information of amino acids?
Response: Thank you for the comment. Radial basis function (RBF) networks are chosen for their local response representations, which are adept at capturing the nuanced spatial features within proteins. The localized sensitivity of RBF units, combined with their collective capacity to approximate complex functions, makes them well-suited for modeling the intricate spatial relationships in protein structures. Moreover, RBF networks provide high computational efficiency and enhanced interpretability, with clearly defined functional areas and weights for each unit, which is crucial for biological applications. The decision to employ an RBF neural network was also influenced by its ability to model spatial distributions through the Gaussian-like function, offering a smooth and flexible approach to represent spatial distances and interactions. The kernel attention mechanism further refines this by emphasizing significant features. We have detailed these advantages and our comparative analysis with other methods in Supplemental Method S6.

Comment 5: The manuscript conducts extensive hyperparameter ablation experiments, but some training parameters, such as the learning rate, are not mentioned. Have detailed experimental settings, parameter information, and the code for model training and evaluation been provided?
Response: Thank you for the comment. The learning rate was set to 0.0001 with the Adam optimizer used for training. Dropout rates were set to 0.5, and batch size was 256. Detailed experimental settings, including the learning rate schedules and optimization strategies, are provided in Supplemental Method S7 and our GitHub introductions. The code for model training and evaluation is available at the GitHub repository: https://github.com/Wssduer/GraphRBF.

Comment 6: Could you discuss the limitations of GraphRBF, such as challenges related to computational resources, model complexity, and data dependency? Furthermore, could you propose future research directions, such as improving the model's spatial feature extraction capability and incorporating more biological knowledge to enhance the model's generalization ability?
Response: Thank you for the comment. The multi-level geometric deep learning model of GraphRBF, although performing excellently, imposes high demands on computational resources due to its complex architecture. A large amount of GPU power and a long turn about one day for full training cycle are required to support model training and inference, which is highly challenging to achieve in resource-limited environments. The complexity of the GraphRBF model is not only reflected in its multi-level structure but also in its requirement for high-dimensional input features. In order to address model complexity, future operations may involve simplifying the structure through techniques like pruning or knowledge distillation, optimizing hyperparameter tuning, and enhancing spatial feature extraction capabilities for better generalization. In future researches, new models can utilize the entire protein structures to improve the spatial feature extraction capability. In addition, future models may integrate more biological knowledge, such as protein functional domains or protein-protein interaction networks, to improve interpretability and biological relevance. The above discussions are added in the Discussion section of the revised manuscript.

Recommendation: In summary, this paper presents an innovative and effective model, GraphRBF, which significantly enhances the accuracy and interpretability of protein binding site predictions. By addressing the reviewer's questions and incorporating the suggested modifications, the paper will be further strengthened. Given the novelty and practical value of this work, I recommend that it be accepted for publication after the necessary major revisions are made.

Lastly, we would like to express our gratitude once again for your valuable comments!
==
Reviewer2：
Overall: The idea behind the deep learning model used in this paper is sound, and the model framework is clearly described both in text and figures. Necessary comparisons with other models using metrics such as

Recall (Rec), F1-Score, and AUC are provided. The authors have made their data sources reliable and have open-sourced the code on GitHub along with a web server. However, the following points need to be clarified.
Web Server: They note on the GraphRBF server at http://liulab.top/GraphRBF/server mentions a fast mode, but I did not find this mode available. Also, I wonder what GeoNet refers to.
Response: Thank you for the comment and we apologize for any inconvenience caused to your use. About the design of the "fast mode", to save users' time of generating PSSM and HMM files, we developed a "fast" version called GraphRBF-fast, which requires only a protein PDB file as input. The "fast" version also performs very well (see Table 1 of the revised manuscript) and has been deployed on our online service. About the availability of the "fast mode", our web server administrator told us that she was recently maintaining and testing the online server and turned off the "fast mode" during that time. Now, she reopens the "fast mode" and you can find it. About GeoNet, the proposed GraphRBF model was named GeoNet before, which was then renamed GraphRBF to better represent the framework. The web server usage introductions have been carefully reorganized to remove any mistakes and make them clearer for users. In addition, we included the comparison metrics of the fast mode in Table 1 of the Results section for comparison with other methods. Thank you very much for the valuable comment.

Dataset: The dataset size is in the range of 10^2 to 10^3, which seems small for the complex task of protein docking. Can such a limited dataset yield a sufficiently generalized result?
Response: Thank you for the comment. There seems a misunderstanding when you are reading the manuscript. The proposed GraphRBF model is not to solve the task of protein docking which takes a protein and another molecule(s) as input. Similar to ScanNet, GraphBind, etc, GraphRBF is designed to predict protein binding residues with other biomolecules (DNA, RNA, or protein) with only the target protein as input, which is a different but critical and challenging prediction task of proteins. Therefore, each residue is treated as a sample and the numbers of samples are 332,659, 537,392, and 369,513, respectively for the protein-DNA, protein-RNA, and protein-protein datasets in this study. And hence, these datasets should be enough to yield generalized results.

Model Framework: Does GraphRBF require both protein sequence and structure as input, or it can work with just one of these? If it requires both, how does it handle prediction tasks when the protein structure is missing? The models compared in the paper, such as DRNApred and TargetDNA, use only sequences, while GraphBind uses only structures. If GraphRBF uses just sequences or structures alone, would it still achieve similar performance?
Response: Thank you for the comment. Just like GraphBind, our model also uses only protein structures, and protein sequences are only employed to extract residue type and residue evolutionary information. For the missing of protein structure, users are suggested to first apply AlphaFold or other related tools to generate a protein structure based on a protein sequence, and then handle prediction tasks by using our tool. The protein structure is essential for extracting features such as DSSP and spatial coordinates, which are critical for constructing the local graph and coordinate system. In the absence of a protein structure, our tool cannot be executed as it fundamentally relies on the three-dimensional coordinates. Since our tool uses only protein structures, like GraphBind, the showed performance of GraphRBF is just based on the running of it using structures alone.

Model Performance:
1.How do the metrics of GraphRBF compare to those of advanced models like AlphaFold3 in the tasks described in the paper?
Response: Thank you for the comment. AlphaFold3 is really strong in predicting the structure of protein complexes with different molecules, which belongs to the prediction task of protein docking. Different from them, the GraphRBF model is designed to predict protein binding residues with other biomolecules (DNA, RNA, or protein) with only the target protein as input, which is a different but critical and challenging prediction task of proteins. Therefore, it is difficult to compare GraphRBF with models like AlphaFold3. Instead, we compared it with advanced methods of the same kind, such as ScanNet, Masif, GraphBind, etc.

2. Accuracy is an intuitive and important metric for evaluating the performance of deep learning models. Although basic, it should be included in the table comparing different models.
Response: Thank you for the comment. We added the Accuracy metric in Table 1 of the revised manuscript and results showed that GraphRBF exhibits the highest Accuracy across all the three datasets.

3. Have the newly predicted binding sites for the SARS-CoV-2 spike protein been confirmed by existing research, or have they been assessed for reliability using other bioinformatics methods, such as docking methods?
Response: Thank you for the comment. We applied GraphRBF to predict binding sites using the unbound spike protein structure 7TGW, and all the predicted binding sites for the SARS-CoV-2 spike protein are validated as follows. (1) We directly assess those predicted binding sites by using known experimentally resolved antigen-antibody complexes. Results show that a large amount of predicted binding sites successfully align with BCEs, particularly for nanobodies (see “Applying GraphRBF to exploring the SARS-CoV-2 spike protein” of the Results section for details). (2) For the predicted binding sites that can not be confirmed by known antigen-antibody complexes, we searched for related literature that reports residue binding information of the protein and results show that many predicted binding sites in regions such as NTD, RBD, SD1, and SD2, are consistent with literature-reported binding residues or regions, including the 'supersite' and other significant sites like Y489 and F486. (3) We made corresponding structural and sequence analyses to further validate the reliability of the remaining predicted sites and found that the residue spatial distributions, the hydrophobic values, and the electrostatic distributions of a predicted region are almost identical with those of two known binding regions, based on which we infer that this predicted binding region has great potential for binding to nanobodies. (4) There are still two predicted regions for which we have not yet collected information to prove whether they are binding regions or not. Considering that the first region is located in the RBD region, it is also very likely that additional relevant binding site information may be identified in future studies. And the second region is close to the S2 region related to immune escape and transmembrane action, which also requires further experimental verification to determine its function.
Lastly, we would like to express our gratitude once again for your valuable comments!
