# Supplementary material for: Protein–protein and protein–nucleic acid binding site prediction via interpretable hierarchical geometric deep learning
Source: Gigascience. 2024 Nov 1;13:giae080. doi: 10.1093/gigascience/giae080 (PMC11528319; doi:10.1093/gigascience/giae080)
Supplement: giae080_Reviewer_1_Report_Original_Submission — Henrik Toft Simonsen -- 6/19/2024 Reviewed [file giae080_reviewer_1_report_original_submission.pdf]

Reviewer Report

Title: Protein-protein and protein-nucleic acid binding site prediction via interpretable hierarchical geometric deep learning

Version: Original Submission **Date:** 8/5/2024

Reviewer name: Jianmin Wang

Reviewer Comments to Author:

This paper introduces a novel hierarchical geometric deep learning model, GraphRBF, designed to predict protein-protein and protein-nucleic acid binding sites. The model demonstrates significant improvements over existing state-of-the-art methods in terms of accuracy and interpretability. The detailed experiments and thorough data analysis provided in the manuscript convincingly showcase the efficacy and novelty of the proposed model. However, I have some serious concerns. Please see below for details.Comment 1: Could you specify the tools and parameters used for extracting atomic features, secondary structure features, and evolutionary information? How are these features integrated into the model inputs? During the feature extraction process, were any feature selection or dimensionality reduction techniques employed? If so, please describe these steps and the methods used in detail. Additionally, please provide a comprehensive overview of the data format inputted into the model.Comment 2: What criteria were used to select the radius and the number of neighbors when constructing the local graph? How do these parameters affect the model's performance?Comment 3: Could you elaborate on the details of constructing the local coordinate system? For instance, when selecting the first direction vector and the second direction vector, were other possible choices considered?Comment 4: The manuscript mentions numerous advantages of the radial basis function model. Was there a specific analysis or explanation provided for these advantages? Additionally, what considerations led to the choice of using a radial basis function neural network to extract spatial distribution information of amino acids?Comment 5: The manuscript conducts extensive hyperparameter ablation experiments, but some training parameters, such as the learning rate, are not mentioned. Have detailed experimental settings, parameter information, and the code for model training and evaluation been provided?Comment 6: Could you discuss the limitations of GraphRBF, such as challenges related to computational resources, model complexity, and data dependency? Furthermore, could you propose future research directions, such as improving the model's spatial feature extraction capability and incorporating more biological knowledge to enhance the model's generalization ability?Recommendation: In summary, this paper presents an innovative and effective model, GraphRBF, which significantly enhances the accuracy and interpretability of protein binding site predictions. By addressing the reviewer's questions and incorporating the suggested modifications, the paper will be further strengthened. Given the novelty and practical value of this work, I recommend that it be accepted for publication after the necessary major revisions are made.

Level of Interest

Please indicate how interesting you found the manuscript: Choose an item.

Quality of Written English

Please indicate the quality of language in the manuscript: Choose an item.

Declaration of Competing Interests

Please complete a declaration of competing interests, considering the following questions:

- Have you in the past five years received reimbursements, fees, funding, or salary from an organisation that may in any way gain or lose financially from the publication of this manuscript, either now or in the future?
- Do you hold any stocks or shares in an organisation that may in any way gain or lose financially from the publication of this manuscript, either now or in the future?
- Do you hold or are you currently applying for any patents relating to the content of the manuscript?
- Have you received reimbursements, fees, funding, or salary from an organization that holds or has applied for patents relating to the content of the manuscript?
- Do you have any other financial competing interests?
- Do you have any non-financial competing interests in relation to this paper?

If you can answer no to all of the above, write 'I declare that I have no competing interests' below. If your reply is yes to any, please give details below.

I declare that I have no competing interests

I agree to the open peer review policy of the journal. I understand that my name will be included on my report to the authors and, if the manuscript is accepted for publication, my named report including any attachments I upload will be posted on the website along with the authors' responses. I agree for my report to be made available under an Open Access Creative Commons CC-BY license (http://creativecommons.org/licenses/by/4.0/). I understand that any comments which I do not wish to be included in my named report can be included as confidential comments to the editors, which will not be published.

Choose an item.

To further support our reviewers, we have joined with Publons, where you can gain additional credit to further highlight your hard work (see: https://publons.com/journal/530/gigascience). On publication of this paper, your review will be automatically added to Publons, you can then choose whether or not to claim your Publons credit. I understand this statement.

Yes Choose an item.
