# Supplementary material for: Protein–protein and protein–nucleic acid binding site prediction via interpretable hierarchical geometric deep learning
Source: Gigascience. 2024 Nov 1;13:giae080. doi: 10.1093/gigascience/giae080 (PMC11528319; doi:10.1093/gigascience/giae080)
Supplement: giae080_Reviewer_2_Report_Original_Submission — Wei Sun -- 7/1/2024 Reviewed [file giae080_reviewer_2_report_original_submission.pdf]

Reviewer Report

Title: Protein-protein and protein-nucleic acid binding site prediction via interpretable hierarchical geometric deep learning

Version: Original Submission **Date:** 8/7/2024

Reviewer name: Ming Chen

Reviewer Comments to Author:

Overall: The idea behind the deep learning model used in this paper is sound, and the model framework is clearly described both in text and figures. Necessary comparisons with other models using metrics such as Recall (Rec), F1-Score, and AUC are provided. The authors have made their data sources reliable and have open-sourced the code on GitHub along with a web server. However, the following points need to be clarified.Web Server: They note on the GraphRBF server at http://liulab.top/GraphRBF/server mentions a fast mode, but I did not find this mode available. Also, I wonder what GeoNet refers to.Dataset: The dataset size is in the range of 10^2 to 10^3, which seems small for the complex task of protein docking. Can such a limited dataset yield a sufficiently generalized result?Model Framework: Does GraphRBF require both protein sequence and structure as input, or it can work with just one of these? If it requires both, how does it handle prediction tasks when the protein structure is missing? The models compared in the paper, such as DRNApred and TargetDNA, use only sequences, while GraphBind uses only structures. If GraphRBF uses just sequences or structures alone, would it still achieve similar performance?Model Performance:1. How do the metrics of GraphRBF compare to those of advanced models like AlphaFold3 in the tasks described in the paper?2. Accuracy is an intuitive and important metric for evaluating the performance of deep learning models. Although basic, it should be included in the table comparing different models.3. Have the newly predicted binding sites for the SARS-CoV-2 spike protein been confirmed by existing research, or have they been assessed for reliability using other bioinformatics methods, such as docking methods?

Level of Interest

Please indicate how interesting you found the manuscript: Choose an item.

Quality of Written English

Please indicate the quality of language in the manuscript: Choose an item.

Declaration of Competing Interests

Please complete a declaration of competing interests, considering the following questions:

- Have you in the past five years received reimbursements, fees, funding, or salary from an organisation that may in any way gain or lose financially from the publication of this manuscript, either now or in the future?
- Do you hold any stocks or shares in an organisation that may in any way gain or lose financially from the publication of this manuscript, either now or in the future?
- Do you hold or are you currently applying for any patents relating to the content of the manuscript?
- Have you received reimbursements, fees, funding, or salary from an organization that holds or has applied for patents relating to the content of the manuscript?
- Do you have any other financial competing interests?
- Do you have any non-financial competing interests in relation to this paper?

If you can answer no to all of the above, write 'I declare that I have no competing interests' below. If your reply is yes to any, please give details below.

I declare that I have no competing interests

I agree to the open peer review policy of the journal. I understand that my name will be included on my report to the authors and, if the manuscript is accepted for publication, my named report including any attachments I upload will be posted on the website along with the authors' responses. I agree for my report to be made available under an Open Access Creative Commons CC-BY license (http://creativecommons.org/licenses/by/4.0/). I understand that any comments which I do not wish to be included in my named report can be included as confidential comments to the editors, which will not be published.

Choose an item.

To further support our reviewers, we have joined with Publons, where you can gain additional credit to further highlight your hard work (see: https://publons.com/journal/530/gigascience). On publication of this paper, your review will be automatically added to Publons, you can then choose whether or not to claim your Publons credit. I understand this statement.

Yes Choose an item.
